# Longitudinal Associations of Sedentary Behavior and Physical Activity With Sleep Duration and Quality in Individuals Living With and Beyond Colorectal Cancer

**DOI:** 10.1177/10732748251397676

**Published:** 2025-11-14

**Authors:** Koen G. Frenken, Laurien M. Buffart, Martijn Bours, Stéphanie O. Breukink, Maryska Janssen-Heijnen, Joop Konsten, Eric T. P. Keulen, Annemone Siffels, Annemarie Koster, Kenneth Meijer, Frank A.J.L. Scheer, Karen Steindorf, Judith de Vos Geelen, Matty P. Weijenberg, Eline H. van Roekel

**Affiliations:** 1 Department of Epidemiology, GROW Research Institute for Oncology and Reproduction, 568601 Maastricht University, Maastricht, the Netherlands; 2 Department of Medical BioSciences, 6034 Radboud University Medical Center, Nijmegen, the Netherlands; 3 Department of Surgery, GROW Research Institute for Oncology and Reproduction, NUTRIM Research Institute of Nutrition and Translational Research in Metabolism, Maastricht University Medical Centre+, Maastricht, the Netherlands; 4 Department of Clinical Epidemiology, 8187 VieCuri Medical Centre, Venlo, the Netherlands; 5 Department of Surgery, 8187 VieCuri Medical Centre, Venlo, the Netherlands; 6 Department of Internal Medicine and Gastroenterology, 159205 Zuyderland Medical Centre Sittard-Geleen, Geleen, the Netherlands; 7 IQ Health Science Department, 6034 Radboud University Medical Center, Nijmegen, the Netherlands; 8 Department of Social Medicine, CAPHRI Care and Public Health Research Institute, 5211 Maastricht University, Maastricht, the Netherlands; 9 Department of Nutrition and Movement Sciences, NUTRIM Research Institute of Nutrition and Translational Research in Metabolism, 685312 Maastricht University, Maastricht, the Netherlands; 10 Division of Sleep Medicine, 1811 Harvard Medical School, Boston, MA, USA; 11 Medical Chronobiology Program, Division of Sleep and Circadian Disorders, Departments of Medicine and Neurology, Brigham and Women’s Hospital, Boston, MA, USA; 12 Division of Physical Activity, Prevention and Cancer, German Cancer Research Center (DKFZ), Heidelberg, Germany; 13 Department of Internal Medicine, Division of Medical Oncology, GROW Research Institute for Oncology and Reproduction, 568601 Maastricht University Medical Centre, Maastricht, the Netherlands

**Keywords:** colorectal cancer survivorship, sedentary behavior, physical activity, sleep duration, sleep quality, nocturnal rest

## Abstract

**Introduction:**

Poor sleep quality and disturbances are common in colorectal cancer (CRC) survivors. However, the impact of sedentary behavior, standing, and physical activity on sleep duration and quality remains under-explored in this population. Therefore, this study examined longitudinal associations of sedentary behavior, standing, and physical activity with sleep outcomes up to 5 years post-treatment.

**Methods:**

A prospective cohort study was conducted among 401 survivors of stage I-III CRC, with repeated measures up to 60 months post-treatment. Thigh-worn accelerometers were used to measure sedentary time (per 2h/day), standing (per 1h/day), and various levels of physical activity (per 1h/day). Sleep duration (h/night) and variability (0%–100%; higher variability equals more sleep disruption) were determined from self-reported sleep times. Sleep quality and insomnia symptoms were assessed using the PSQI and EORTC questionnaires, respectively, where higher scores indicated worsened symptoms. Longitudinal associations were analyzed using confounder-adjusted linear mixed models.

**Results:**

Total mean sedentary time was 10.3 ± 1.8 h/day, mean standing time was 2.9 ± 1.2 h/day, mean total physical activity was 1.5 ± 0.6 h/day, and mean sleep duration was 8.7 ± 1.0 h/night at 6 weeks post-treatment. More sedentary behavior was longitudinally associated with a shorter sleep duration (β = −0.28; 95%CI = −0.35, −0.21). More standing was associated with a shorter sleep duration (β = −0.21; 95%CI = −0.25, −0.16), higher sleep variability (β = 0.29%; 95%CI = 0.03, 0.55), improved sleep quality (β = −0.24; 95%CI = −0.43, −0.04), and reduced insomnia symptoms (β = −2.00; 95%CI = −3.27, −0.72). More total physical activity was associated with a shorter sleep duration (β = −0.31; 95%CI = −0.41, −0.21) and higher sleep variability (β = 0.59%; 95%CI = 0.11, 1.10). More moderate-to-vigorous physical activity was associated with a shorter sleep duration (β = −0.04; 95%CI = −0.07, −0.00) and reduced insomnia symptoms (β = −1.74; 95%CI = −2.69, −0.79).

**Conclusion:**

Our findings suggest that physical (in)activity and standing are relevant for sleep-related symptoms in CRC survivors. Future studies should examine whether substituting sedentary behavior with standing and/or physical activity may alleviate sleep-related symptoms in CRC survivors.

## Plain Language Summary

How sitting and physical activity can affect sleep in people living with and beyond colorectal cancer: Introduction: Poor sleep quality is common among people living with and beyond colorectal cancer. However, the effect of sitting, standing, and physical activity on sleep duration and quality has not been studied extensively. Therefore, this study investigated how sitting and physical activity affect sleep in people living with and beyond colorectal cancer over a period of 5 years after their treatment ended. Methods: 401 survivors of colorectal cancer were included within this study. These survivors were measured repeatedly over a period of 5 years. We used a device worn on the thigh to measure how much time people spent sitting, standing, and moving. Additionally, we measured how many hours people spent sleeping, how their sleep quality was, and whether people had trouble falling asleep using diaries and questionnaires. Results: After treatment, people who spent more time sitting, standing, and being physically active had a shorter sleep duration. In addition, people who spent more time standing had improved sleep quality and less trouble falling asleep. We also observed that people who spent time moving at a higher intensity experienced less trouble falling asleep. Conclusion: These results indicate that survivors who stand and move more experience less sleep problems and have less trouble falling asleep. Therefore, there may be a critical role for these behaviours in improving sleep in survivors of colorectal cancer. More research is needed to investigate the effects of substituting sitting by more time standing and being physically active.

## Introduction

Worldwide, colorectal cancer (CRC) is one of the most common cancer types, with approximately 2 million new cases and it is the second most common cause of cancer deaths.^1^ Advances in treatment and earlier detection have led to a growing number of CRC survivors (5-year survival rate between 81%–92% for stage I-III).^2^ Largely due to aging, the incidence of CRC is expected to rise to 3.2 million in 2040, additionally contributing to the increasing number of survivors.^3^ Many survivors experience long-term symptoms related to cancer and/or its treatment, such as fatigue (30%–60%) and insomnia (30%–75%), which can significantly impact quality of life.^4-6^ Insomnia symptoms, affecting 30%–75% of newly diagnosed or recently treated cancer patients (mixed types), are characterized by persistent poor sleep quality, difficulty falling or staying asleep, despite having suitable conditions for rest, and may lead to daytime dysfunction.^5,7,8^ Self-reported sleep quality is an individual’s self-satisfaction with all aspects of the sleep experience including sleep efficiency, sleep latency, sleep duration, and wake after sleep onset.^9^ The prevalence of insomnia in patients undergoing chemotherapy for various cancers was found to be 3 times higher compared to healthy individuals (of similar ages), and has been shown to negatively affect quality of life in cancer survivors.^10^ Results from previous studies in healthy populations showed optimal sleep duration as a curvilinear relationship, with 7-9 hours being optimal.^11,12^ A sleep duration of less than 7 hours has been linked with adverse health outcomes such as diabetes, hypertension, heart disease, depression and increased risk of mortality, while sleeping more than 9 hours is also associated with increased health risks.^11,12^ Hence, finding strategies to optimize sleep duration and quality in cancer patients who experience sleep problems is vital.

Numerous randomized controlled trials (RCTs) in healthy populations have demonstrated that interventions improving physical activity and reducing sedentary behavior improved sleep duration and quality.^13-15^ Many cancer survivors have low levels of physical activity and spend approximately 8.2-10.8 hours per day sedentary, which is higher than the general population,^16,17^ with about 0.5-3.1 more hours per day.^18^ However, the associations between physical activity and sleep in cancer populations remain inconclusive, and associations for standing time with sleep outcomes are unknown. Several RCTs, primarily involving walking,^19-23^ have reported small to modest improvements in sleep quality, while a systematic review found no effects on sleep measures.^24^ In contrast, a scoping review on sleep disturbances in cancer populations indicated that physical activity interventions improved sleep behavior, reduced sleep disturbances, and enhanced sleep quality in patients with CRC.^25^ A previous cross-sectional study in cancer survivors (including CRC) found that higher physical activity and higher levels of sedentary behavior decreased the likelihood of reporting less than 8 hours of sleep.^26^

To the best of our knowledge, no studies have examined the longitudinal associations of sedentary behavior, standing and physical activity behaviors with sleep duration, quality and insomnia in CRC survivors. Therefore, this study investigated how sedentary behavior, standing and physical activity are associated with sleep duration and quality up to 5 years after treatment. We hypothesized that high levels of sedentary behavior and low levels of physical activity were longitudinally associated with suboptimal sleep duration (<7 or >9 hours/night) and reduced sleep quality in survivors of CRC.

## Methods

### Study Design and Population

This study utilized data from the Energy for life after ColoRectal cancer (EnCoRe) study, an ongoing prospective cohort of survivors of stage I-III CRC in the south of the Netherlands (Registered under no. NL6904 at https://www.onderzoekmetmensen.nl/en). Since the 18^th^ of April 2012, patients newly diagnosed at 3 hospitals (Maastricht University Medical Center+, VieCuri Medical Center, and Zuyderland Medical Center), were enrolled. Eligibility criteria included 18 years or older, stage I-III CRC, living in the Netherlands, speaking and understanding Dutch, and having no comorbidities that could hinder participation (ie, no visual impairments). Repeated measurements were performed by trained personnel at participants’ homes at diagnosis, 6 weeks (baseline), 6 months, 12 months, 24 months, and 60 months post-treatment. Inclusion and follow-up measurements of patients were paused between March and October 2020 because of the COVID-19 pandemic. Follow-up measurements were conducted remotely via postal service in combination with detailed written instructions and follow-up phone calls with additional instructions (conducted via post: n = 2 for 6 months, n = 15 for 12 months, n = 32 for 24 months and n = 78 for 60 months). From July 2022 onwards, all measurements were resumed at the participants’ homes by trained personnel except for 60 month post-treatment measurement which remained remote. Data collected up to October 1^st^, 2023, were used for this manuscript. For the current analysis, we excluded participants without accelerometer data and/or potential confounding variables (see statistical analysis) at all post-treatment time points.

**Figure 1.**
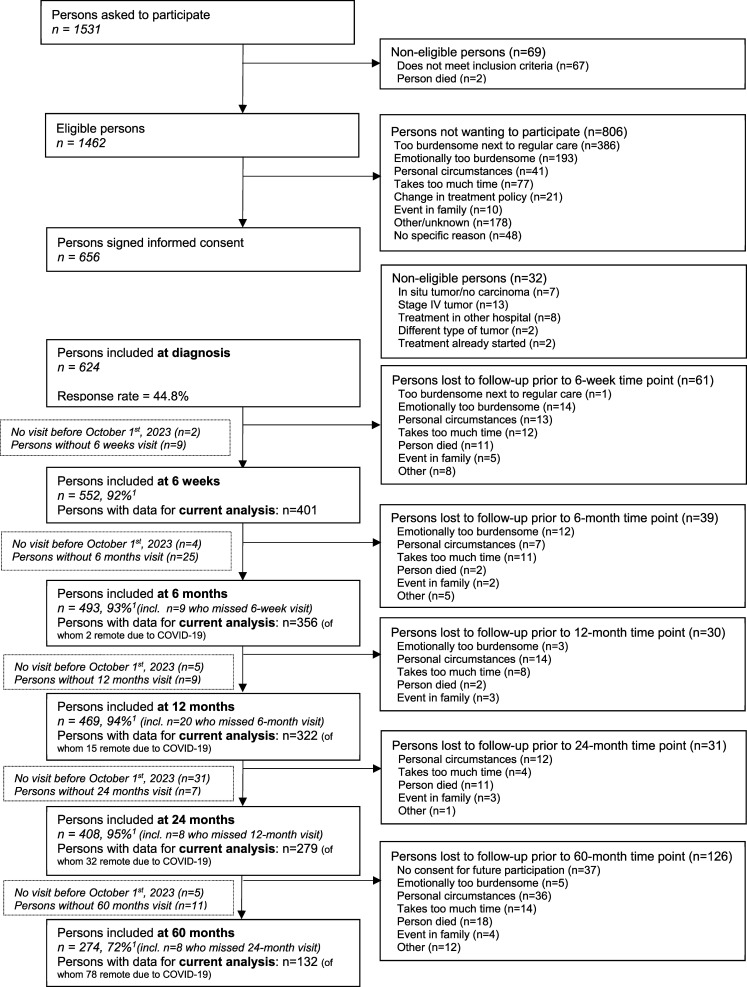
Flow Diagram of the Inclusion of Participants Within the EnCoRe Study and the Number of Post-treatment Measurements Included in the Analyses Presented in This Paper. Data of Home Visits Performed before October 1st, 2023 Were Included in the Analyses. ^1^Response Rate = (Persons With Home visits)/(Persons With Home visits + persons Lost to Follow-up – Persons Died). The Declining Number of Participants at Subsequent Time Points is Mainly because not all Participants Included at Diagnosis From April 2012 Onwards had Reached These Time Points in October 2023

Consequently, a total of 401 participants were included in the current analysis at 6 weeks post-treatment (response rate: 92%), and 356 at 6 months (93%), 322 at 12 months (94%), 279 at 24 months (95%) and 132 at 60 months post-treatment (72%). The decreasing absolute numbers are largely due to participants not having reached all post-treatment follow-up time points at the time of data acquisition in October 2023. A flow diagram describing patient inclusion within the EnCoRe study and the number of measurements included in the current analysis can be found in Figure 1.

The EnCoRe study was approved by the Medical Ethics Committee of the University Hospital Maastricht and Maastricht University (METC 11-3-075) on February 29^th^, 2012. All participants provided written informed consent.

This study is reported according to the STROBE guidelines.^27^

### Objectively Assessed Sedentary Behavior, Standing and Physical Activity Parameters

Time spent in sedentary behavior, standing, and physical activity were objectively assessed using the validated tri-axial MOX activity monitor (MMOXX1, upgraded version of the CAM; Maastricht Instruments B.V., Maastricht, The Netherlands).^28^ The accelerometer was worn on the right anterior upper thigh for 7 consecutive days (24 hours/day) at each post-treatment time point. The accelerometer recorded raw accelerations in 3 orthogonal sensor axes at a sampling rate of 25 Hz. Accelerometer data were processed using a custom MATLAB program (version R2022a; The MathWorks, Inc., Natick, MA). For these analyses, data on hours/day of total sedentary behavior and prolonged sedentary behavior (uninterrupted bouts of sedentary behavior, ie, sitting or lying (including potential naps) during waking hours at a low intensity of ≤1.5 METs of at least 30 minutes), were used.^29-32^ Standing time was defined as time spent upright during self-reported waking hours with an energy expenditure of ≤1.5 METs.^31,33^ Total physical activity was defined as any movement or posture during self-reported waking hours exceeding 1.5 METs.^34^ Additionally, the dichotomy index (I<O) was quantified as the proportion of in-bed (I) activity counts that are less than the median out-of-bed (O).^35^ Lower values demonstrate more day-night disruption and weaker behavioral activity rhythms while higher values for the I<O indicate more robust rhythms.^35^ For the sedentary behavior, standing and total physical activity variables, a measurement day was considered valid if it included at least 10 hours of valid wear time.^36^ All variables were calculated for each valid day and averaged across all available days at each post-treatment time point. For the dichotomy index, 24 hours of valid wear time was required (ie, with no non-wear periods).^35^ For this analysis, only data from post-treatment measurements with at least 4 valid days were included. 99.6% of all measurements also included at least 1 weekend day.

The MOX accelerometer has demonstrated moderate to high reproducibility and high validity for measuring sedentary behavior, standing and total physical activity.^37^ Its reliability in differentiating between various intensity levels of physical activity (ie, light-intensity physical activity and moderate to vigorous physical activity) is limited,^38^ and therefore only total PA was determined with the MOX accelerometer.

### Self-Reported Physical Activity

The validated Short QUestionnaire to ASsess Health-enhancing physical activity (SQUASH) was used to determine self-reported time spent in light-intensity physical activity (LPA) and moderate-to-vigorous physical activity (MVPA).^39^ Participants reported time spent on commuting, household, work, and leisure time activities over a week. Each activity was assigned a MET value using Ainsworth’s Compendium of Physical Activities.^40^ Total weekly LPA and MVPA (hours/week) were calculated by summing the time spent on activities with energy expenditures of 1.5-3.0 METs and >3.0 METs, respectively.^34^ This was subsequently divided by 7 to achieve hours per day of total weekly LPA and MVPA. The SQUASH has demonstrated moderate reliability (test–retest: Spearman’s ρ = 0.57-0.58),^41^ and a relative validity that is comparable to other physical activity questionnaires when compared to accelerometer data (Spearman’s ρ = 0.40 for moderate-intensity activities).^41^

### Sleep Duration, Variability and Coefficient of Midpoint of Sleep

Sleep duration and variability were based on self-reported sleep times within 7-day sleep/food diaries. Participants were asked to register daily, the times they went to lie down in bed and got out of bed. This was subsequently visually checked with the MOX accelerometer graphs and if registered times were missing MOX times were recorded. There would be a maximum of 6 valid nights possible since the accelerometer was worn for 7 days (so the first wake up on day 1 and the last evening on day 7 were discarded). A minimum of 4 valid nights were used. Based on these self-reported times, sleep durations were calculated based on time in bed for each day in hours/night. Sleep duration was averaged across all valid (according to collected accelerometer data) measurement nights. The coefficient of sleep variation (0%-100%) was calculated as the standard deviation (SD) of the mean sleep duration per day divided by the mean sleep duration, the ratio was subsequently multiplied by 100.^42^ A greater variability indicates a more disrupted sleep rhythm while a lower variability indicates a more stable sleep rhythm.

The coefficient of variation of midpoint of sleep (in hours), ie, midpoint between sleep onset and waking up,^43^ was determined by dividing the SD of the midpoint of sleep of the valid nights by the average midpoint of sleep across all valid nights and was multiplied by 100. A lower coefficient is indicative of a more regular timing of sleep while a higher coefficient indicates more variable timing of sleep.

### Self-Reported Sleep Quality and Insomnia Symptoms

The Pittsburgh Sleep Quality Index (PSQI) is a self-rated questionnaire which assesses sleep quality and disturbances over 1 month.^44^ The total PSQI assesses sleep quality and disturbances in 7 different components namely subjective sleep quality, sleep latency, sleep duration, sleep efficiency, sleep disturbance, use of sleep medication, and daytime dysfunction which together sum to a maximum of 21 points for the overall global PSQI score. For the current paper only the PSQI global score was used in the main analysis. Higher scores indicate worse sleep quality while lower scores indicate better sleep quality.^45^ The PSQI has been shown to have strong reliability and validity as determined by the Kirshner and Guyatt framework.^46^ Additionally the PSQI was found to have moderate structural validity.^45^ The PSQI questionnaire was added to the study measurements approximately 5 years after the start of the EnCoRe study, resulting in fewer available measurements (see Table 2 for exact numbers).

Insomnia symptoms were measured with the insomnia subscale of the European organization for research and treatment of cancer Core Quality of Life Questionnaire (EORTC QLQ-C30).^47^ Participants were asked whether they experience sleep disturbances “not at all”, “a little”, “quite a bit”, or “very much”. This answer is subsequently transformed to a 0-100 scale. Higher scores indicate worsened insomnia symptoms. A study comparing the Jenkins Sleep Scale (JSS) and the single-item sleep scale of the EORTC-QLQ-C30 found that the JSS only detected sleep problems marginally better while finding that the EORTC-QLQ-C30 is sufficient for investigations conducted on a group level.^48^

### Sociodemographic, Lifestyle and Clinical Covariables

Sociodemographic characteristics, including age (years), sex (male/female) and education level (low, medium or high; see Table 1 for details) were self-reported at diagnosis. Smoking status (current, former or never), employment status (employed/unemployed), partner status (yes/no), and the presence of a stoma were also self-reported at diagnosis and during each post-treatment measurement. Body mass index (BMI; kg/m^2^) was calculated at diagnosis and at post-treatment time points based on measurements taken by researchers or by self-reported height (only at diagnosis) and weight (all measurement time points). From October 2020 onwards, due to the COVID-19 pandemic, anthropometric measurements were switched to self-reported measurements. BMI was categorized according to the categories defined by the World Health Organization (WHO): underweight (BMI <18.5 kg/m^2^), normal weight (18.5 ≤ BMI <25 kg/m^2^), overweight (25 ≤ BMI <30 kg/m^2^), and obesity (BMI ≥30 kg/m^2^).^49^ Dietary intake recorded via a 7-day food diary at each post-treatment time point was used to determine alcohol consumption (g/day). The food diary also allowed patients to self-report naps taken at various time points throughout the day, which was recoded into a dichotomous value (yes/no). Sleep medication use (was reported as not used, less than once per month, 1-2 times per month and more than 3 times per month) was recorded through the PSQI questionnaire and dichotomized (yes/no sleep medication use). Comorbidities (0, 1 or ≥2) were assessed using the 13-item Comorbidity Questionnaire at each post-treatment timepoint.^50^ Clinical data, obtained from medical records, included cancer stage (I, II, or III), tumor site (colon, rectosigmoid, or rectum) and treatment type (surgery, chemotherapy (neoadjuvant/adjuvant), and/or radiotherapy).

**Table 1.** Sociodemographic, Lifestyle, and Clinical Characteristics of the Study Population of Survivors of Colorectal Cancer at All Post-treatment Time Points

Characteristics	6 weeks post- treatment (n = 401)^a^	6 months post- treatment (n = 356)^a,b^	12 months post- treatment (n = 322)^a,b^	24 months post- treatment (n = 279)^a,b^	60 months post- treatment (n = 132)^a,b^
Age (years), mean ± SD	67.7 ± 9.3	67.6 ± 9.3	67.7 ± 9.1	69.4 ± 8.8	70.9 ± 8.9
Sex (male), *n* (%)	266 (66.3)	231 (64.9)	214 (66.5)	187 (67.0)	82 (62.1)
Education level, *n* (%)^c^					
Low	103 (25.7)	92 (25.8)	76 (23.6)	69 (24.7)	25 (18.9)
Medium	153 (38.2)	140 (39.3)	128 (39.8)	111 (39.8)	56 (42.4)
High	145 (36.2)	124 (34.8)	118 (36.7)	99 (35.5)	51 (38.6)
BMI (kg/m^2^), mean ± SD	27.4 ± 4.4	28.1 ± 4.5	28.4 ± 4.6	28.4 ± 4.7	27.5 ± 4.7
BMI categorical, *n* (%)					
Underweight (<18.5 kg/m^2^)	2 (0.5)	0 (0.0)	1 (0.3)	1 (0.4)	2 (1.5)
Healthy weight (18.5 – 24.9 kg/m^2^)	130 (32.4)	92 (25.8)	76 (23.6)	71 (25.5)	43 (32.6)
Overweight (25.0 – 29.9 kg/m^2^)	166 (41.4)	158 (44.4)	138 (42.9)	116 (41.f6)	48 (36.4)
Obese (≥30 kg/m^2^)	103 (25.7)	106 (29.8)	107 (33.2)	91 (32.6)	39 (29.6)
Smoking status, *n* (%)					
Current	31 (7.7)	28 (7.9)	29 (9.0)	21 (7.5)	11 (8.3)
Former	241 (60.1)	220 (61.8)	198 (61.5)	180 (64.5)	89 (67.4)
Never	129 (32.2)	108 (30.3)	95 (29.5)	78 (28.0)	32 (24.2)
Partner (yes), n (%)	323 (80.6)	283 (79.5)	259 (80.4)	218 (78.1)	101 (76.5)
Employment (yes), *n* (%)	118 (29.4)	103 (28.9)	95 (29.5)	62 (22.6)	28 (21.2)
Stoma (yes), *n* (%)	101 (25.2)	53 (14.9)	46 (14.3)	41 (14.7)	18 (13.6)
Treatment, *n* (%)					
Surgery (yes)	357 (89.0)	322 (90.4)	287 (89.1)	250 (89.6)	113 (85.6)
Chemotherapy (yes)	149 (37.2)	130 (36.5)	119 (37.0)	105 (37.6)	43 (32.6)
Radiotherapy (yes)	91 (22.7)	80 (22.4)	74 (23.0)	64 (22.9)	34 (25.8)
Tumor site, *n* (%)					
Colon	262 (65.3)	238 (66.9)	210 (65.2)	182 (65.2)	80 (60.6)
Rectosigmoid & rectum	139 (34.7)	118 (33.2)	112 (34.8)	97 (34.8)	52 (39.4)
Cancer stage, *n* (%)					
Stage I	121 (30.2)	115 (32.3)	103 (32.0)	89 (31.9)	42 (31.8)
Stage II	107 (26.7)	87 (24.4)	78 (24.2)	70 (25.1)	37 (28.0)
Stage III	173 (43.1)	154 (43.3)	141 (43.8)	120 (43.0)	53 (40.2)
Comorbidities, *n* (%)					
0	80 (20.0)	85 (23.9)	82 (25.5)	68 (24.4)	40 (30.3)
1	103 (25.7)	94 (26.4)	77 (23.9)	71 (25.5)	35 (26.5)
≥2	218 (54.4)	177 (49.7)	163 (50.6)	140 (50.2)	57 (43.2)

Abbreviations: BMI, body mass index; SD, standard deviation.

^a^Percentages may not add up to 100% due to rounding.

^b^The decreasing absolute numbers are largely since participants had not yet reached all post-treatment follow-up time points at the time of data acquisition in October 2023.

^c^Highest attained education, low: no education, primary education, or basic vocational education; medium: advanced vocational education or senior secondary vocational education; high: senior secondary general education, higher professional education, or academic higher education.

### Statistical Analysis

Descriptive statistics were used to summarize sociodemographic, lifestyle, and clinical characteristics, as well as sedentary behavior, standing time, physical activity, I<O, and sleep parameters at each post-treatment time point. Normally distributed quantitative variables were presented as mean ± SD, while non-normally distributed variables as well as variables where mean values were not informative, were presented as median with inter-quartile range (IQR). Categorical variables were described using frequencies and percentages. Pearson’s correlation coefficients were calculated to determine the correlation between all exposures and outcomes at 6 weeks post-treatment to gain insight into interrelationships.

An a-priori power calculation for longitudinal analyses was performed.^51^ With an expected sample size of 259, 246, 230, and 202 at 6 months, 1 year, 2 years and 5 year post-treatment respectively, and assuming an alpha of 5% and a power of 80%, we calculated the smallest detectable contrast in outcome variables to be 1.0 for the PSQI, 5.8-6.1 for the insomnia subscale of the EORTC, and 0.2-0.3 hours for sleep duration for each time points. All of these were smaller than the minimal important contrast for the respective outcome scores, ie, 1.7, 9.0 and 0.6 hours, respectively, indicating that our study was sufficiently powered to detect relevant associations in our population.^52,53^

Linear mixed models were used to analyze longitudinal associations of sedentary behavior and physical activity with sleep parameters from 6 weeks to 60 months post-treatment. Total sedentary time and prolonged sedentary time were analyzed in 2-hour increments, while standing time, total physical activity, LPA and MVPA were analyzed in 1-hour increments per day, approximating the SD of these variables. The dichotomy index was standardized by dividing individual values by the mean SD across all 5 post-treatment time points, and modelled per 1 SD.

The relationship between the exposures and sleep duration was modeled using linear mixed models for sleep duration as continuous measures and using longitudinal multinomial logistic regression with sleep duration categorized into 3 categories: less than 7 hours of sleep, between 7 and 9 hours of sleep and more than 9 hours of sleep, where 7-9 hours of sleep was the reference category. This model was used since based on literature a curvilinear relationship could also be expected as well between sedentary behavior and physical activity variables with sleep duration. The resulting odds ratios (OR) represent the likelihood of being in a certain category of sleep duration (eg, <7 hours) relative to the reference category (7-9 hours of sleep), per unit change in physical activity or sedentary behavior variables (eg, 2 hours/day more sedentary behavior).

Longitudinal models were adjusted for potential confounders, predefined based on existing literature. Fixed (time-invariant) confounders included age at enrollment (continuous; years), sex neoadjuvant therapy (chemo- and/or radiotherapy: yes/no), adjuvant chemotherapy (yes/no), and education level (low, medium, high). Time-variant confounders, measured at all post-treatment time points, included number of comorbidities (0, 1, ≥2), BMI (continuous; kg/m^2^), stoma (yes/no), smoking status (current, former, never), partner status (yes/no), employment status (employed, unemployed/retired), alcohol intake (continuous; g/day), and time since the end of treatment (continuous; days). A likelihood-ratio test was used to determine whether adding a random slope would improve model fit. In addition to overall longitudinal associations, inter- and intra-individual associations were analyzed separately. Inter-individual associations (average differences between participants over time) were estimated by adding centered person mean values to the model, while intra-individual associations (within-participant changes over time) were estimated by modeling individual deviations from the person-mean value.^54^

Effect modification by sex, BMI (continuous per measurement moment), and time since end of treatment (continuous per 6 months), was examined by including interaction terms in the models. Significance was set at *P* < 0.05 for interaction terms.

To gain insight into the possible direction of longitudinal associations, we conducted a sensitivity analysis using time lag models. In these models, sedentary behavior and physical activity variables at earlier time points were paired with sleep quality and duration parameters at subsequent time points reflecting a more natural direction of associations. Additional sensitivity analyses included adjustment for potential additional confounders, including presence of napping during the day (yes/no), presence of sleep medication as measured by the PSQI (yes/no), alcohol intake (g/day) and cancer stage (I, II or III). Additionally, in another sensitivity analysis, all sedentary variables and LPA were adjusted for MVPA. In another model MVPA and all sedentary variables were adjusted for LPA. Furthermore, a sensitivity analysis was conducted, adjusting for whether participants were measured remotely due to the COVID-19 pandemic. Moreover, we performed a sensitivity analysis additionally adjusting for standing time. Lastly, due to the smaller sample size at 60 months, we performed a sensitivity analysis up to 24 months post-treatment to investigate whether the lower response rate at 60 months vs the other follow-up time-points influenced our findings. All statistical analyses were performed using Stata 16.0 (StataCorp LLC) with statistical significance set at *P* < 0.05 (two-sided). Sensitivity analyses results were presented as comparison plots created in R-Studio version 2024.09.0 + 375.

## Results

### Study Population Characteristics

In total, 401 patients were included. The average age of participants at 6 weeks post-treatment was 67.7 (SD = 9.3) years and 66.3% was male (Table 1). 74.4% of the participants were medium-to-highly educated, 80.6% of participants had a partner and 29.4% were employed. The most common diagnosis was colon cancer (65.3%) while the remaining participants (34.7%) were diagnosed with rectosigmoid or rectum cancer. The most common treatment was surgery (89.0%), 37.2% received chemotherapy and 22.7% received radiotherapy. More than half of the participants had 2 or more comorbidities (54.4%).

### Descriptives of Sedentary Behavior, Physical Activity and Sleep Variables

At the baseline for longitudinal analyses (ie, 6 weeks post-treatment), total sedentary behavior was 10.3 h/day (SD: 1.8 h/day), standing was 2.9 h/day (SD: 1.2 h/day), and total physical activity was 1.5 h/day (SD: 0.6 h/day). Sleep duration was 8.7 h/night (SD: 1.0 h/night) and PSQI score was 5.4 points (SD: 3.3 points). Further descriptives over time regarding physical activity and sedentary behavior variables as well as sleep variables can be found in Table 2 and baseline Pearson correlation coefficients between all exposure and outcome variables are presented in Supplemental Figure 1.

**Table 2.** Descriptive Analyses of Sedentary Behavior, Physical Activity and Sleep Variables in the Study Population of Survivors of Colorectal Cancer

Characteristics	6 weeks post-treatment (N = 401)	6 months post-treatment (N = 356)^a^	12 months post-treatment (N = 322)^a^	24 months post-treatment (N = 279)^a^	60 months post-treatment (N = 132)^a^
Sedentary behavior and physical activity variables (mean ± SD)/(median – 25^th^ percentage – 75^th^ percentage) (hours/day)					
Total sedentary behavior	10.3 ± 1.8	10.1 ± 1.6	9.9 ± 1.8	9.9 ± 1.6	9.9 ± 1.8
Prolonged sedentary behavior^b^	4.9 ± 2.3	4.4 ± 1.9	4.3 ± 1.9	4.5 ± 2.0	4.5 ± 2.0
Standing	2.9 ± 1.2	3 .3 ± 1.1	3.3 ± 1.1	3.3 ± 1.1	3.2 ± 1.0
Total physical activity	1.5 ± 0.6	1.7 ± 0.6	1.6 ± 0.6	1.6 ± 0.6	1.6 ± 0.7
Light physical activity	1.1 (0.3 – 2.5)	1.5 (0.6 – 3.0)	1.5 (0.4 – 3.1)	1.5 (0.5 – 3.0)	1.5 (0.6 – 3.2)
Moderate-to-vigorous activity	1.0 (0.5 – 2.0)	1.4 (0.8 – 2.4)	1.4 (0.7 – 2.4)	1.4 (0.8 – 3.9)	1.4 (0.8 – 2.5)
Dichotomy index^c^	0.9 (0.8 – 0.9)	0.9 (0.8 – 0.9)	0.9 (0.8 – 0.9)	0.9 (0.8 – 0.9)	0.9 (0.8 – 0.9)
Sleep parameters (mean ± SD)/(median – 25^th^ percentage – 75^th^ percentage)					
Sleep duration^d^	8.7 ± 1.0	8.7 ± 1.0	8.6 ± 1.0	8.6 ± 1.0	8.6 ± 0.9
Sleep duration variability^d^	7.1 (4.6 – 10.7)	7.5 (5.2 – 11.0)	7.8 (5.2 – 11.1)	7.9 (4.7 – 10.9)	6.3 (4.3 – 9.1)
Midpoint of sleep variability^d^	10.3 (6.5 – 16.3)	12.3 (7.7 – 18.5)	11.4 (7.4 – 18.2)	10.4 (7.0 – 16.5)	9.9 (6.4 – 14.0)
Sleep quality^e^ *(PSQI)*	5.4 ± 3.3	5.1 ± 3.4	5.1 ± 3.4	5.0 ± 3.3	6.5 ± 2.9
Insomnia *(EORTC)*	21.9 ± 27.7	18.5 ± 26.6	17.5 ± 25.3	16.7 ± 24.3	15.4 ± 23.8

Abbreviations: SD, standard deviation; IQR, interquartile range.

^a^The decreasing absolute numbers are largely due to the fact that participants had not yet reached all post-treatment follow-up time points at the time of data acquisition in October 2023.

^b^Uninterrupted bouts of sedentary behavior, ie, sitting or lying during waking hours at a low intensity of ≤1.5 METs of at least 30 minutes.

^c^The dichotomy index was only calculated for days without non-wear; therefore, there were fewer measurements available for this parameter at 6 weeks post-treatment (n = 375), at 6 months post-treatment (n = 333), at 12 months post-treatment (n = 309), at 24 months post-treatment (n = 267), and at 60 months post-treatment (n = 128).

^d^Sleep duration, sleep duration variability and midpoint of sleep variability were based on food diaries and checked by the MOX data, in some cases sleep information was missing therefore slightly fewer measurements were available for this parameter at 6 weeks post-treatment (n = 394), at 6 months post-treatment (n = 349), at 12 months post-treatment (n = 316), at 24 months post-treatment (n = 268), and at 60 months post-treatment (n = 130).

^e^The PSQI was introduced into the EnCoRe study in 2017, 5 years after the start of the study. Therefore, fewer measurements were available for this parameter at 6 weeks post-treatment (n = 146), at 6 months post-treatment (n = 128), at 12 months post-treatment (n = 131), at 24 months post-treatment (n = 173), and at 60 months post-treatment (n = 126).

### Longitudinal Associations of Sedentary Behavior and Physical Activity with Sleep Duration and Quality

#### Sedentary behavior and sleep parameters

Increased sedentary behavior was statistically significantly associated over time with a shorter sleep duration (Table 3). For each 2-hour increase per day in sedentary behavior, sleep duration decreased on average −0.28 hours per night (95% CI: −0.35, −0.21). Associations with sleep duration were driven by both intra-individual (β = −0.20; 95%CI = −0.26, −0.14) as well as inter-individual (β = −0.37; 95%CI = −0.47, −0.26) associations. Prolonged sedentary behavior (per 2 hours/day) was significantly associated with a lower coefficient of variation of midpoint of sleep, only for the inter-individual analyses (β = −0.86%; 95%CI = −1.73, −0.00).

**Table 3.** Longitudinal Associations of Sedentary Behavior and Physical Activity With Self-Reported Sleep Parameters Between 6-Weeks and 60 -Months Post-treatment in the Study Population of Survivors of Colorectal Cancer

		Sleep duration (Mean sleep duration in hours per night; h/night)		Coefficient of sleep variation (Variation in sleep duration; 0%–100%)		Coefficient of midpoint of sleep (Variation in midpoint of sleep; 0%–100%)		Sleep quality (PSQI global score; 0-21 points)		Insomnia (EORTC-QLQ-C30; 0-100 score)	
		β^a^	95% CI	β^a^	95% CI	β^a^	95% CI	β^a^	95% CI	β^a^	95% CI
Total sedentary behavior (2h/day) *(MOX)*	Adjusted^c,d^	**−0.28**	**(**−**0.35,** −**0.21)**	0.11	(−0.26, 0.48)	0.27	(−0.43, 0.98)	0.09	(−0.13, 0.31)	−0.11	(−1.66, 1.45)
	Intra^e^	**−0.20**	**(**−**0.26,** −**0.14)**	−0.11	(−0.53, 0.31)	−0.32	(−1.40, 0.77)	0.06	(−0.19, 0.32)	−0.34	(−2.24, 3.05)
	Inter^f^	**−0.37**	**(**−**0.47,** −**0.26)**	−0.44	(−0.08, 0.95)	0.20	(−0.67, 1.06)	0.19	(−0.27, 0.65)	0.36	(−2.32, 3.05)
Prolonged sedentary behavior (2h/day)^g^ *(MOX)*	Adjusted^c,d^	−0.06	(−0.11, 0.00)	−0.08	(−0.34, 0.21)	−0.39	(−1.17, 0.39)	0.12	(−0.10, 0.35)	0.00	(−1.42, 1.43)
	Intra^e^	−0.04	(−0.10, 0.02)	−0.07	(−0.48, 0.34)	−0.37	(−1.23, 0.49)	0.17	(−0.10, 0.45)	0.65	(−1.24, 2.54)
	Inter^f^	−0.08	(−0.17, 0.01)	−0.10	(−0.52, 0.32)	**−0.86**	**(**−**1.73,** −**0.00)**	0.02	(−0.35, 0.39)	−0.84	(−3.00, 1.31)
Standing behavior (h/day) *(MOX)*	Adjusted^c,d^	**−0.21**	**(**−**0.25,** −**0.16)**	**0.29**	**(0.03, 0.55)**	0.50	(−0.04, 1.05)	**−0.24**	**(**−**0.43,** −**0.04)**	**−2.00**	**(**−**3.27,** −**0.72)**
	Intra^e^	**−0.18**	**(**−**0.24,** −**0.13)**	0.14	(−0.23, 0.51)	**0.81**	**(0.06, 1.56)**	−0.08	(−0.32, 0.16)	−1.04	(−2.72, 0.63)
	Inter^f^	**−0.26**	**(**−**0.34,** −**0.18)**	**0.43**	**(0.07, 0.80)**	0.17	(−0.61, 0.95)	**−0.54**	**(**−**0.87,** −**0.20)**	**−3.30**	**(**−**5.26,** −**1.34)**
Total physical activity (h/day) *(MOX)*	Adjusted^c,d^	**−0.31**	**(**−**0.41,** −**0.21)**	**0.59**	**(0.11, 1.10)**	**2.41**	**(1.21, 3.61)**	−0.26	(−0.64, 0.12)	−1.92	(−4.32, 0.47)
	Intra^e^	**−0.30**	**(**−**0.40,** −**0.20)**	0.49	(−0.21, 1.19)	**1.65**	**(0.21, 3.08)**	−0.29	(−0.78, 0.20)	−1.55	(−4.76, 1.65)
	Inter^f^	**−0.31**	**(**−**0.45,** −**0.16)**	**0.69**	**(0.02, 1.35)**	**2.13**	**(0.74, 3.53)**	−0.22	(−0.83, 0.39)	−2.38	(−5.94, 1.17)
Light-intensity physical activity (h/day) *(Self-reported SQUASH)*	Adjusted^c,d^	**−0.07**	**(**−**0.10,** −**0.05)**	0.05	(−0.10, 0.20)	**0.39**	**(0.07, 0.70)**	−0.06	(−0.18, 0.06)	−0.66	(−1.41, 0.08)
	Intra^e^	**−0.06**	**(**−**0.09,** −**0.03)**	0.13	(−0.06, 0.33)	**0.69**	**(0.29, 1.09)**	−0.04	(−0.18, 0.11)	−0.17	(−1.07, 0.73)
	Inter^f^	**−0.10**	**(**−**0.16,** −**0.05)**	−0.08	(−0.32, 0.16)	−0.12	(−0.62, 0.39)	−0.11	(−0.31, 0.10)	**−1.67**	**(**−**2.94,** −**0.39)**
Moderate-to-vigorous-intensity physical activity (h/day) *(Self-reported SQUASH)*	Adjusted^c,d^	**−0.04**	**(**−**0.07,** −**0.00)**	−0.06	(−0.25, 0.13)	0.04	(−0.36, 0.45)	−0.12	(−0.28, 0.04)	**−1.74**	**(**−**2.69,** −**0.79)**
	Intra^e^	−0.03	(−0.07, 0.01)	−0.20	(−0.46, 0.06)	0.05	(−0.49, 0.59)	−0.14	(−0.34, 0.05)	**−2.03**	**(**−**3.23,** −**0.83)**
	Inter^f^	−0.05	(−0.12, 0.01)	0.11	(−0.18, 0.40)	0.04	(−0.57, 0.64)	−0.08	(−0.35, 0.20)	−1.25	(−2.80, 0.30)
Dichotomy index^b^ *(MOX)*	Adjusted^c,d^	−0.03	(−0.07, 0.00)	−0.08	(−0.31, 0.15)	−0.10	(−0.37, 0.57)	0.02	(−0.15, 0.19)	−0.85	(−1.93, 0.23)
	Intra^e^	−0.03	(−0.07, 0.01)	−0.06	(−0.32, 0.20)	−0.04	(−0.57, 0.50)	0.09	(−0.10, 0.27)	−0.46	(−1.66, 0.73)
	Inter^f^	−0.05	(−0.15, 0.04)	−0.13	(−0.58, 0.33)	0.52	(−0.43, 1.47)	−0.33	(−0.73, 0.08)	**−2.46**	**(**−**4.88,** −**0.41)**

Values in bold are statistically significant (*P* < 0.05).

Abbreviations: PSQI: Pittsburgh Sleep Quality Index, EORTC: European Organization for Research and Treatment of Cancer, SQUASH: Short Questionnaire to Assess Health-enhancing physical activity.

^a^The β-coefficients indicate the overall longitudinal difference in the outcome score using linear mixed models per 2 hours increase in sedentary behavior variables, per 1 hour increase in physical activity variables or per 1 SD increase in the dichotomy index.

^b^The dichotomy index was included in the model per standard deviation (SD) increase, being the average SD across time points.

^c^Linear mixed-models were adjusted for sex (male/female), age at enrollment (years), time since end of treatment (days), neo-adjuvant chemotherapy and/or radiotherapy (yes/no), adjuvant therapy chemotherapy (yes/no), comorbidities (0, 1, ≥2), BMI (kg/m^2^), stoma (yes/no), smoking (former, current, never), employment status (yes/no), education level (low/medium/high) and partner status (yes/no).

^d^A random slope was added to the model when the model improved statistically significantly using a likelihood-ratio test.

^e^The β-coefficients indicate the average change in the outcome score over time when total sedentary behavior and prolonged sedentary increases with 2 hours per day or when standing, total physical activity, LPA and MVPA increase with 1 hour per day or when the dichotomy index increases per 1 SD between time points from 6 weeks to 60 months post-treatment within individuals.

^f^The β-coefficients indicate the average difference in the outcome score between individuals differing when total sedentary behavior and prolonged sedentary is 2 hours per day higher or when standing, total physical activity, LPA MVPA is 1 hour per day higher or when the dichotomy index is 1 SD higher between time points from 6 weeks to 60 months post-treatment.

^g^Uninterrupted bouts of sedentary behavior, ie, sitting or lying during waking hours at a low intensity of ≤1.5 METs of at least 30 minutes.

When sleep duration was analyzed as a categorical outcome (Table 4), more sedentary behavior was associated with a higher odds of having a shorter sleep duration <7 hours/night (OR per 2 hours/day more total sedentary behavior: 1.87; 95%CI = 1.11, 3.15), compared to having a normal sleep duration of 7-9 hours/night. More total sedentary behavior and prolonged sedentary behavior were also associated with a significant lower odds of having a longer sleep duration of >9 hours/night (OR: 0.38; 95%CI = 0.29, 0.50 and 0.70; 95%CI = 0.56, 0.88, respectively), compared to having a normal sleep duration of 7-9 hours/night.

**Table 4.** Longitudinal Associations of Sedentary Behavior and Physical Activity Parameters With Sleep Duration Between 6-Weeks and 60 -Months Post-treatment in the Study Population of Survivors of Colorectal Cancer

		Sleep duration	
		OR^a^	95% CI of OR
Total sedentary behavior (2h/day) *(MOX)*	<7 hours	**1.87**	**(1.11, 3.15)**
	7-9 hours (REF)		
	>9 hours	**0.38**	**(0.29, 0.50)**
Prolonged sedentary behavior (2h/day)^b^ *(MOX)*	<7 hours	1.30	(0.86, 1.95)
	7-9 hours (REF)		
	>9 hours	**0.70**	**(0.56, 0.88)**
Standing behavior (h/day) *(MOX)*	<7 hours	**2.01**	**(1.33, 3.03)**
	7-9 hours (REF)		
	>9 hours	**0.57**	**(0.46, 0.70)**
Total physical activity (h/day) *(MOX)*	<7 hours	1.34	(0.65, 2.76)
	7-9 hours (REF)		
	>9 hours	**0.41**	**(0.28, 0.61)**
Light-intensity physical activity (h/day) *(Self-reported SQUASH)*	<7 hours	1.07	(0.87, 1.31)
	7-9 hours (REF)		
	>9 hours	**0.71**	**(0.62, 0.81)**
Moderate-to-vigorous-intensity-physical activity (h/day) *(Self-reported SQUASH)*	<7 hours	1.09	(0.83, 1.43)
	7-9 hours (REF)		
	>9 hours	0.96	(0.82, 1.11)
Dichotomy index *(MOX)*	<7 hours	1.29	(0.81, 2.06)
	7-9 hours (REF)		
	>9 hours	0.99	(0.84, 1.18)

Values in bold are statistically significant (*P* < 0.05).

REF, reference; OR, odds ratio; the ranges for the various categories are: category 1 (<7 hours of sleep), category 2 (reference category: 7-9 hours of sleep), category 3 (>9 hours of sleep).

Abbreviations: SQUASH: Short Questionnaire to Assess Health-enhancing physical activity.

Models were adjusted for sex (male/female), age at enrollment (years), time since end of treatment (days), neo-adjuvant chemotherapy and/or radiotherapy (yes/no), adjuvant therapy chemotherapy (yes/no), comorbidities (0, 1, ≥2), BMI (kg/m^2^), stoma (yes/no), smoking (former, current, never), employment status (yes/no), education level (low/medium/high) and partner status (yes/no).

^a^The OR indicates the overall odds of being in each sleep duration category relative to the reference category of a normal sleep duration (7-9 hours of sleep per night) for an increase of 2 hours in sedentary behavior variables, per 1 hour increase in physical activity variables, and per 1 SD increase in the dichotomy index.

^b^Uninterrupted bouts of sedentary behavior, ie, sitting or lying during waking hours at a low intensity of ≤1.5 METs of at least 30 minutes.

#### Standing behavior

More standing was statistically significantly associated with a shorter sleep duration, improved sleep quality, and improved insomnia symptoms (EORTC), and with a higher variation in total sleep duration over time (Table 3). For each hour increase in standing time, the sleep duration decreased with 0.21 hours/night (95% CI = −0.25, −0.16), sleep quality improved with −0.24 points (95% CI = −0.43, −0.04), insomnia symptoms improved by −2 points (95% CI = −3.27, −0.72) and the coefficient of sleep variation increased 0.29% (95%CI = 0.03, 0.55). Associations with sleep duration over time were driven by both intra-individual changes (β = −0.18; 95%CI = −0.24, −0.13) and inter-individual differences (β = −0.26; 95%CI = −0.34, −0.18), while for sleep quality, insomnia and the coefficient of sleep variation, associations were driven mostly by inter-individual associations (Table 3). More standing was also associated with a higher variation in midpoint of sleep, although only intra-individual associations were statistically significant (β = 0.81%; 95%CI = 0.06, 1.56). Increases in standing time were associated with a higher odds of having a shorter sleep duration than 7 hours (OR: 2.01; 95%CI = 1.33, 3.03) and a lower odds of having a longer sleep duration than 9 hours (OR: 0.57; 95%CI = 0.46, 0.70), compared to having a normal sleep duration of 7-9 hours/night (Table 4).

#### Total physical activity

Total physical activity was significantly associated over time with a shorter sleep duration (β = −0.31; 95%CI = −0.41, −0.21), and with a higher coefficient of sleep variation (β = 0.59%; 95%CI = 0.11, 1.10) and coefficient of midpoint of sleep (β = 2.41%; 95%CI = 1.21, 3.61). For the coefficient of sleep variation, associations were only driven by inter-individual differences (for standing: β = 0.43%; 95%CI = 0.07,0.80 and for TPA: 0.69%; 95%CI = 0.02, 1.35) (Table 3). For the remainder of the associations mentioned above, the associations were driven by both intra- and inter-individual differences. Total physical activity was associated with a lower odds of having a longer sleep duration than 9 hours (OR: 0.41; 95%CI = 0.28, 0.61), compared to having a normal sleep duration of 7-9 hours/night (Table 4).

#### Light-intensity-physical activity and moderate-to-vigorous-physical activity

More LPA (hour per day) was significantly associated over time with a decrease in sleep duration of 0.07 hours (95%CI = −0.10, −0.05), which was driven by both intra-individual changes (β = −0.06; 95%CI = −0.09, −0.03) and inter-individual differences (β = −0.10; 95%CI = −0.16, −0.05) (Table 3). Additionally, LPA was associated with an increase in the coefficient of midpoint of sleep variation (β = 0.39%; 95%CI = 0.07, 0.70), driven mainly by intra-individual (β = 0.69%; 95%CI = 0.29, 1.09) associations. Lastly, LPA was associated with a decrease in insomnia symptoms via inter-individual associations (β = −1.67; 95%CI = −2.94, −0.39). More LPA was also associated with a lower odds of having a longer sleep duration than 9 hours (OR: 0.71; 95%CI = 0.62, 0.81), compared to having a normal sleep duration of 7-9 hours/night (Table 4).

MVPA was significantly associated over time with shorter sleep duration (β = −0.04 hours, 95%CI = −0.07, −0.00 per hour MVPA) and less insomnia (β = −1.74; 95%CI = −2.69, −0.79). In multinomial logistic regression analysis, MVPA was not significantly associated with a sleep duration of less than 7 or more than 9 hours/night compared to those with a sleep duration between 7-9 hours.

#### Dichotomy index

A higher dichotomy index was statistically significantly associated only with lower inter-individual differences over time for the insomnia score (Table 3). For every SD increase in the dichotomy index, the insomnia symptom score decreased on average 2.46 points (95%CI = −4.88, −0.41).

### Interaction and Sensitivity Analyses

No significant interactions were found for sex, BMI and time since end of treatment (data not shown). The sensitivity analysis with additional adjustment for stage, napping, LPA, MVPA and alcohol showed similar associations of comparable magnitudes as in the main analysis. Regarding additional adjustments for sleep medication and time lag model, associations were slightly changed in magnitude, but did not change the conclusions for the associations under investigation. (Supplemental Figures 2-8). Regarding the additional adjustment for remote measurements as result of the COVID-19 pandemic, associations were similar with similar magnitudes (data not shown). When adjusting for standing time (Supplemental Tables 11-12), the association between total sedentary time and sleep duration became non-significant, whereas associations for prolonged sedentary behavior with both continuous and categorical sleep duration became significant. Associations for total physical activity also became significant. Lastly, the sensitivity analysis up to 24 months post-treatment revealed no significant changes from the overall associations that were observed up to 60 months post-treatment (Supplemental Tables 9-10).

## Discussion

In this study, we found that higher levels of sedentary time were associated with a shorter sleep duration in CRC survivors. Moreover, more standing was associated with a shorter sleep duration, more variation in sleep duration, better sleep quality and reduced insomnia symptoms. More TPA, more LPA and more MVPA were associated with a shorter sleep duration, while more TPA and LPA were associated with more sleep variation in both duration and midpoint of sleep. With regard to categories of sleep duration, we found that more sedentary time and more standing resulted in a greater odds of sleeping less than 7 hours relative to sleeping 7-9 hours per night. Additionally, more sedentary time, prolonged sedentary behavior, standing, total physical activity and LPA all resulted in lower odds of sleeping more than 9 hours relative to sleeping 7-9 hours per night.

Our findings that both sedentary behavior and physical activity variables are associated with shorter sleep duration is likely related to the 24-hour daily cycle, where more time spent sedentary or active, likely reduces the time available for sleep. These findings align with previous studies suggesting a U-shaped relationship, where individuals who are either highly sedentary or engage in high amounts of physical activity experience shorter sleep durations.^55^ Notably, our multinomial logistic regression models indicate that physical activity likely supports an ‘optimal’ sleep duration of 7-9 hours, which is widely recognized as most favorable.^11,12^ Despite some of these associations being small, together, they can contribute to larger changes in sleep duration. Studies in postmenopausal women and general populations report that high sedentary time as well as high occupational physical activity or MVPA is associated with an increased likelihood of shorter sleep duration.^15,56-58^ Additionally, a previous RCT in women with insomnia found that those who exercised 30 min aerobically, 3 times per week, slept, on average, 15 minutes longer per night.^59^ Contrarily, a meta-analysis further concluded that total sleep time is not significantly affected by physical activity.^60^ Results of previous studies are unable to be directly translated to cancer populations as biological alterations that occur because of the cancer and its treatment are unlike regular sleep issues as well as that cancer populations are generally older and experience more comorbidities than healthy adults. Therefore, there is a lack of research specifically examining these associations and the effects of exercise on sleep duration in cancer populations, highlighting the importance of our work. Additional RCTs should be performed in cancerous populations to examine causality.

Our findings that more time spent standing and in physical activity (including LPA) was associated with more variation in sleep duration and midpoint of sleep is unexpected as regular physical activity has been shown to improve sleep parameters responsible for variability such as sleep efficiency, onset latency and overall rhythmicity.^61^ Higher sleep variation has been linked to adverse health outcomes such as inflammation, adverse metabolic outcomes, and mortality in various populations, including cancer.^62^ The lack of associations in our population might be explained by physical activity being performed too close to bedtime, as some studies have shown impaired sleep after late-night exercise.^63-65^ Additional research should shed light on possible timing of physical activity during the day as well as other potential mechanisms that could explain the increased variation due to physical activity, such as fatigue or non-sleep related medication usage.

Regarding sleep quality, we found that more standing time was associated with better sleep quality. Unlike our findings, a study among high school students found that increased standing did not affect sleep quality.^66^ Other studies have found thar regular physical activity improves sleep quality,^13,60,67^ however our results show a tendency for improved sleep quality with more physical activity, but associations did not reach statistical significance. More research among CRC survivors should be done to assess possible influences on sleep quality via for example, RCTs looking at the effects of standing as well as various forms of physical activity or applying substitution models for various forms of activity.

Our findings that more standing time and higher MVPA levels were associated with fewer insomnia symptoms supports a previous systematic review and meta-analysis in patients with pathological diseases (including mixed cancer types) and a RCT among a population with insomnia, showing positive effects of various structured physical activity plans, and particularly MVPA on reducing insomnia symptoms.^59,68^ Further research in the form of RCTs is needed to support our results within CRC survivors and to conclude directionality and causality.

Strengths of this study are its prospective design and extensive repeated measurements over a 5-year period in a large cohort of survivors of CRC, enabling appropriate control for relevant confounders as well as the ability to differentiate between intra and inter-individual associations. Nevertheless, there are also several limitations to consider. Firstly, despite adjusting for many confounders in our analyses, we still missed information on other potentially relevant confounders (eg, caffeine intake). Secondly, the use of self-reported sleep measures may have introduced reporting bias and hampered distinguishing between sleep duration and time in bed. Third, due to the observational nature of the study, causal relationships cannot be inferred. Fourthly, despite the use of objective physical activity data, the accelerometer used could not distinguish well between different intensities of physical activity and therefore the SQUASH questionnaire was used, which can be prone to recall bias and possible overestimation.^69^ Finally, the response rate of 44.8% at inclusion could have introduced a selection toward participants who were healthier and higher educated who may have been more likely to join the study, while those with poorer health or more complaints might have had greater difficulty participating. Although this may have resulted in a more selective study sample, it may not have influenced the observed associations. The sensitivity analysis and time-lag analysis confirmed that the magnitudes of the associations remained consistent, supporting the robustness of our results While for the sensitivity analysis with an extra adjustment for standing time, some associations between movement behaviors and sleep parameters changed in strength and statistical significance, the direction of associations remained consistent. The changes in effect sizes and strength likely reflect the high intercorrelation between sedentary behavior, standing and physical activity behaviors within the 24-hour period as when 1 increases, another is reduced in time. Future research should investigate a substitution model approach to examine how reallocating time between specific waking behaviors influences sleep duration. Unfortunately, we could not investigate this since information as sleep duration was assessed through another measurement instrument (self-reported questionnaire) than the other measures of activity (objective accelerometer).

## Conclusion

Our findings indicate that in the first 5 years after CRC treatment, less sedentary time as well as more physical activity are associated with reduced sleep duration. Additionally, higher standing and total physical activity are associated with more sleep variation and less insomnia and better sleep quality. Additionally, our findings suggest that physical (in)activity and standing are relevant for sleep-related symptoms in CRC survivors. Future studies should examine whether substituting sedentary behavior with standing and/or physical activity may alleviate sleep-related symptoms in CRC survivors.

## Supplemental Material

Supplemental Material - Longitudinal Associations of Sedentary Behavior and Physical Activity With Sleep Duration and Quality in Individuals Living With and Beyond Colorectal Cancer Supplemental Material for Longitudinal Associations of Sedentary Behavior and Physical Activity With Sleep Duration and Quality in Individuals Living With and Beyond Colorectal Cancer by Koen G. Frenken, Laurien M. Buffart, Martijn Bours, Stéphanie O. Breukink, Maryska Janssen-Heijnen, Joop Konsten, Eric T. P. Keulen, Annemone Siffels, Annemarie Koster, Kenneth Meijer, Frank A.J.L. Scheer, Karen Steindorf, Judith de Vos Geelen, Matty P. Weijenberg, and Eline H. van Roekel in Cancer Control

## Data Availability

Data described in the manuscript, code book, and analytic code will be made available upon request pending (eg, application and approval, payment, other). Requests for data of the EnCoRe study can be sent to Dr Martijn Bours, Department of Epidemiology, GROW Research Institute for Oncology and Reproduction, Maastricht University, the Netherlands (email: m.bours@maastrichtuniversity.nl).

## Appendix

### List of Abbreviations

BMI: Body mass index
CRC: Colorectal cancer
EnCoRe: Energy for Life after Colorectal Cancer
I<O: Dichotomy index
LPA: Light-intensity physical
MET: metabolic equivalence unit
MVPA: Moderate-to-vigorous physical activity
OR: Odds ratio
PSQI: Pittsburg Sleep Quality Index
SD: standard deviation
Sleep med: Sleep medication
SQUASH: Short Questionnaire to Assess Health-Enhancing Physical Activity
WCRF: World Cancer Research Fund
WHO: World Health Organization
