# Supplementary material for: Longitudinal Associations of Sedentary Behavior and Physical Activity With Sleep Duration and Quality in Individuals Living With and Beyond Colorectal Cancer
Source: Cancer Control. 2025 Nov 14;32:10732748251397676. doi: 10.1177/10732748251397676 (PMC12618820; doi:10.1177/10732748251397676)
Supplement: Supplemental Material - Longitudinal Associations of Sedentary Behavior and Physical Activity With Sleep Duration and Quality in Individuals Living With and Beyond Colorectal Cancer [file sj-pdf-1-ccx-10.1177_10732748251397676.pdf]

	SED	PROSED	STAND	TPA	LPA	MVPA	I<O	DUR	PSQI	SDVAR	MIDVAR	INSOM
**SED[1]**	1.00											
**PROSED[1]**	0.66	1.00										
**STAND[1]**	-0.46	-0.43	1.00									
**TPA[1]**	-0.43	-0.62	0.48	1.00								
**LPA[2]**	-0.01	-0.03	0.18	0.05	1.00							
**MVPA[2]**	-0.15	-0.17	0.15	0.45	0.04	1.00						
**I<O[1]**	-0.58	-0.73	0.54	0.62	0.03	0.17	1.00					
**DUR[3]**	-0.27	-0.01	-0.24	-0.15	-0.12	-0.13	-0.05	1.00				
**PSQI[4]**	0.08	0.00	-0.05	-0.09	-0.05	-0.13	-0.04	0.21	1.00			
**SDVAR[3]**	0.13	-0.03	-0.03	0.07	0.00	0.08	-0.01	-0.29	-0.05	1.00		
**MIDVAR[3]**	0.00	-0.13	0.00	0.15	0.10	0.12	0.02	-0.06	-0.04	0.63	1.00	
**INSOM[5]**	0.07	0.02	-0.11	-0.12	-0.02	-0.09	-0.07	0.13	0.80	0.00	0.02	1.00

Supplementary Figure 1. Pearson correlation coefficients at 6 weeks post-treatment between sedentary behavior, physical activity sleep parameters. Color spectrum towards green indicates more positive correlation while more towards red indicates more negative correlation. Abbreviations: SED, sedentary behavior; PROSED, prolonged sedentary behavior; STAND, standing behavior; TPA, total physical activity; LPA, light intensity physical activity; MVPA, moderate- to-vigorous physical activity; I<O, dichotomy index; DUR, Sleep duration; PSQI, Pittsburgh sleep quality index global score; SDVAR, Sleep duration variability; MIDVAR, Midpoint of sleep variability; INSOM, EORTC insomnia scale. [1] Determined by the MOX accelerometer; [1] Determined by the SQUASH questionnaire; [3] Determined by sleep/food diary; [4] Determined by PSQI questionnaire; [5] Determined by EORTC QLQ-C30 insomnia scale.

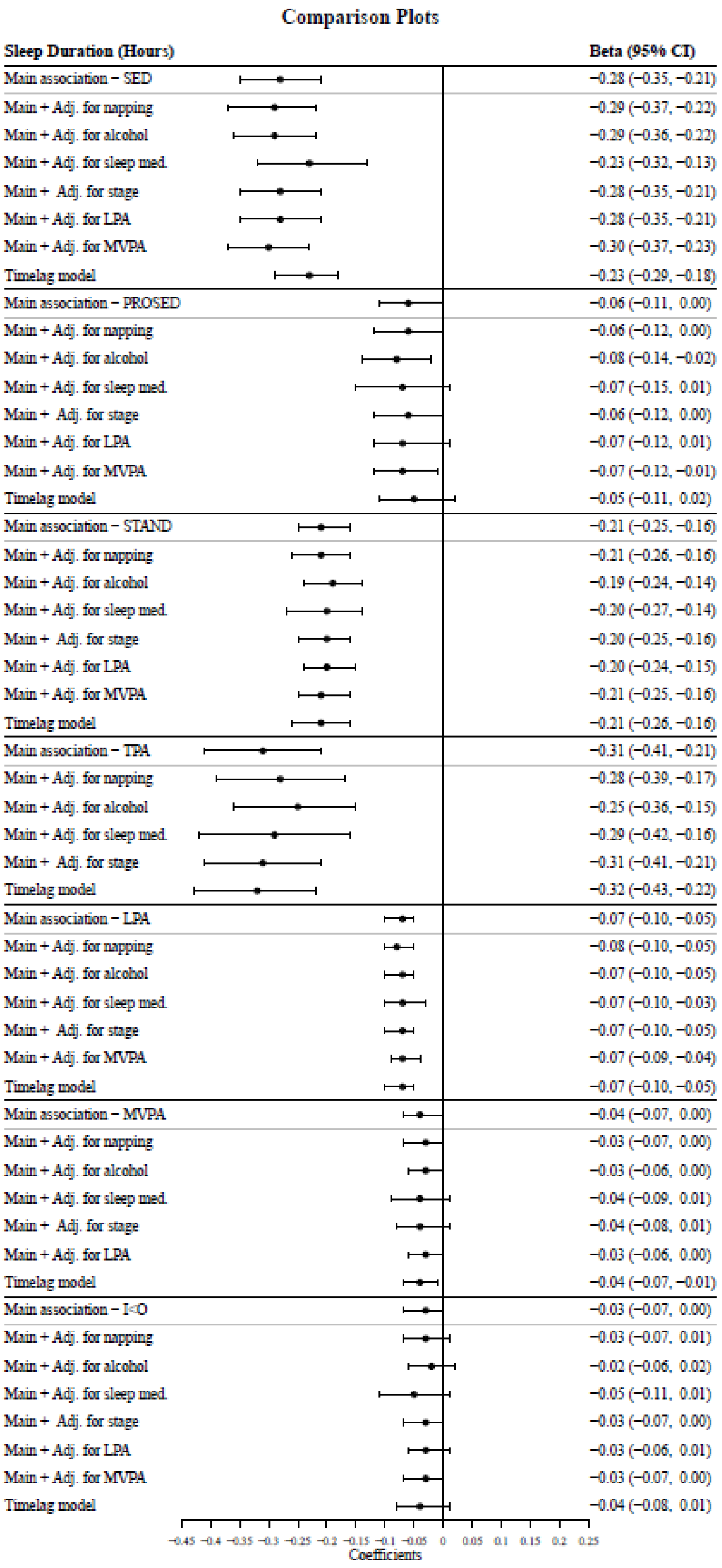

Supplementary Figure 2. Longitudinal adjusted associations of the various sensitivity analysis comparing the main associations with the outcome sleep duration with the additional adjustment to the main model for napping, alcohol, sleep medication, stage, LPA and MVPA. Abbreviations: SED, sedentary behavior; PROSED, prolonged sedentary behavior; STAND, standing behavior; TPA, total physical activity; LPA, light intensity physical activity; MVPA, moderate- to-vigorous physical activity; I<O, dichotomy index.

* Models were already adjusted for sex (male/female), age at enrollment (years), time since end of treatment (days), neo-adjuvant chemotherapy and/or radiotherapy (yes/no), adjuvant therapy chemotherapy (yes/no), comorbidities (0, 1, ≥2), BMI (kg/m^2), stoma (yes/no), smoking (former, current, never), employment status (yes/no), education level (low/medium/high) and partner status (yes/no).

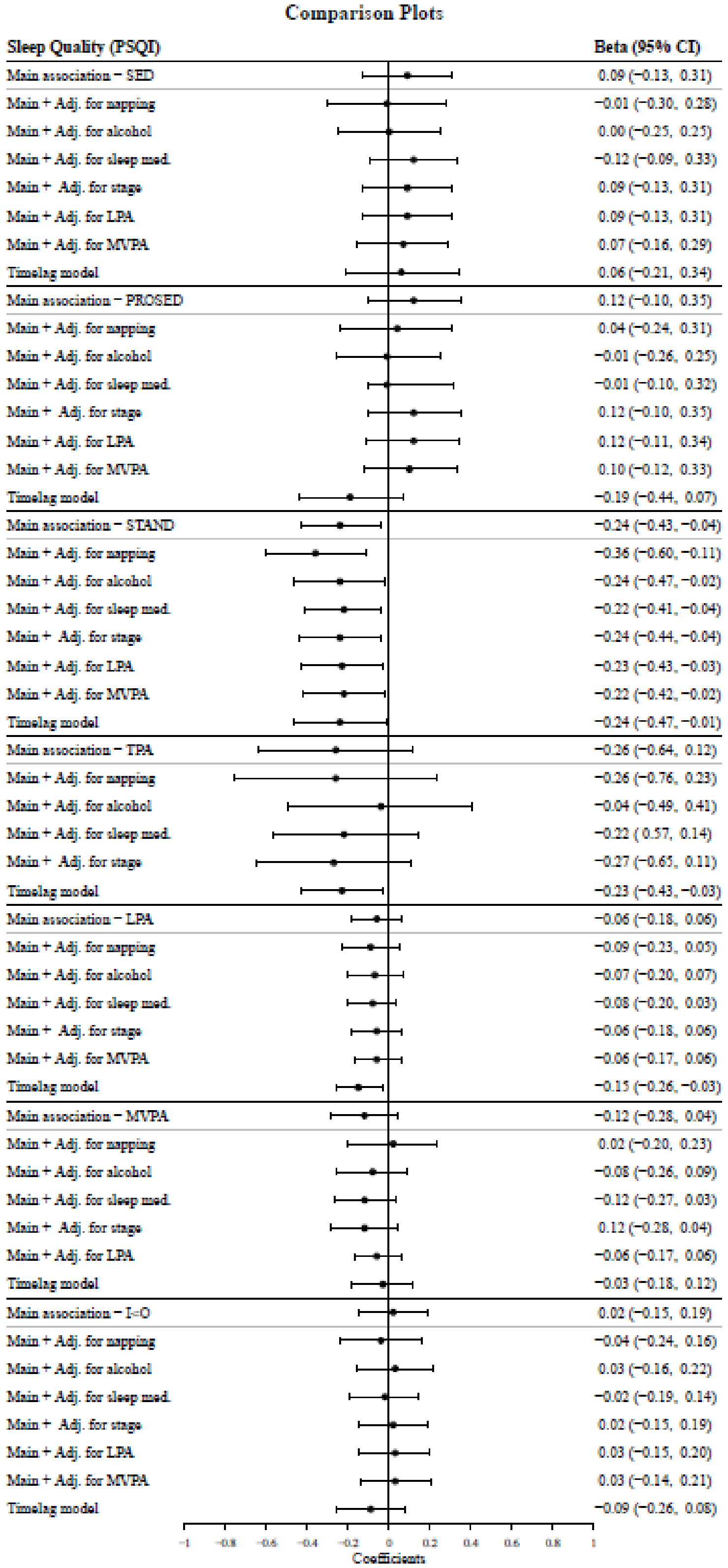

Supplementary Figure 3. Longitudinal adjusted associations of the various sensitivity analysis comparing the main associations with the outcome sleep quality duration with the additional adjustment to the main model for napping, alcohol, sleep medication, stage, LPA and MVPA. Abbreviations: SED, sedentary behavior; PROSED, prolonged sedentary behavior; STAND, standing behavior; TPA, total physical activity; LPA, light intensity physical activity; MVPA, moderate- to-vigorous physical activity; I<O, dichotomy index; PSQI, Pittsburgh Sleep Quality Index.

* Models were already adjusted for sex (male/female), age at enrollment (years), time since end of treatment (days), neo-adjuvant chemotherapy and/or radiotherapy (yes/no), adjuvant therapy chemotherapy (yes/no), comorbidities (0, 1, ≥2), BMI (kg/m^2), stoma (yes/no), smoking (former, current, never), employment status (yes/no), education level (low/medium/high) and partner status (yes/no).

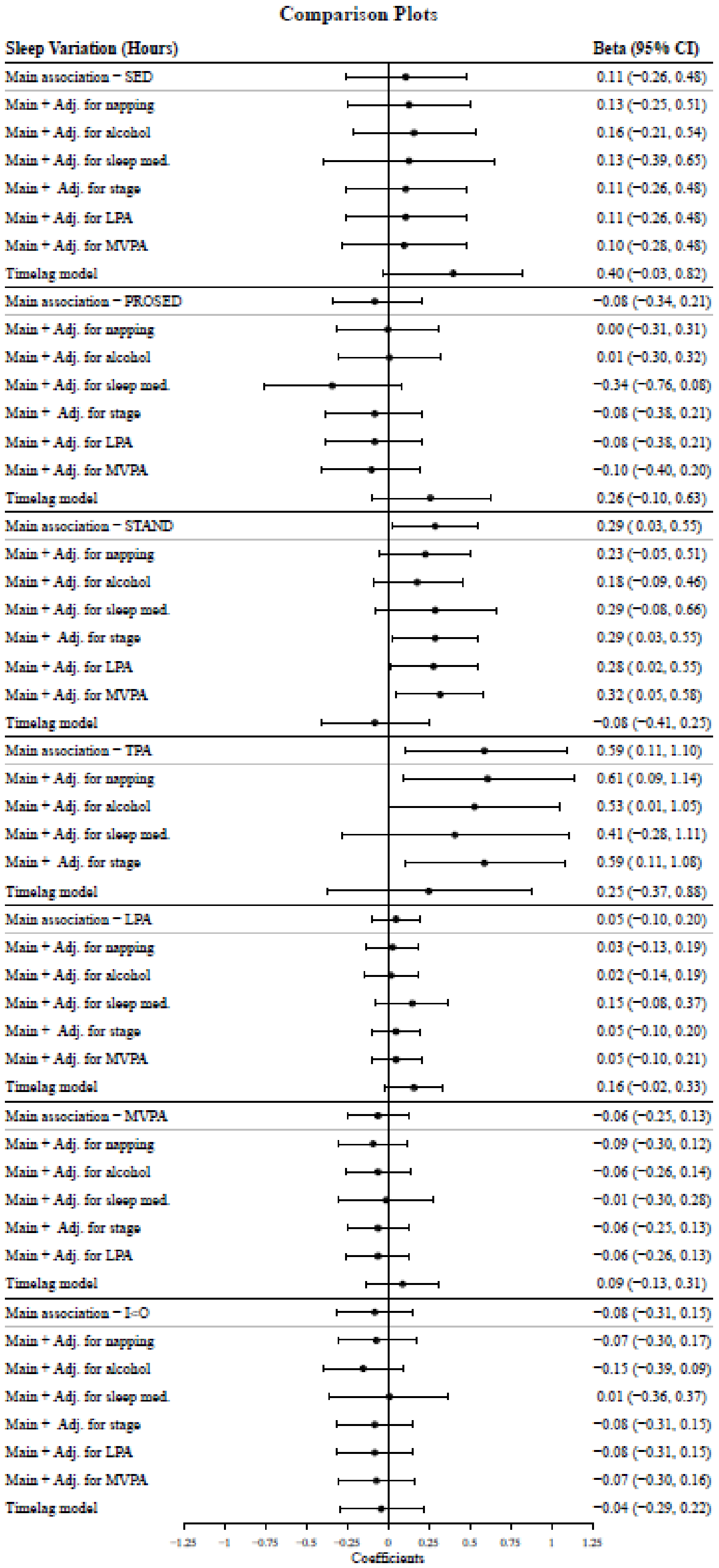

Supplementary Figure 4. Longitudinal adjusted associations of the various sensitivity analysis comparing the main associations with the outcome sleep variation with the additional adjustment to the main model for napping, alcohol, sleep medication, stage, LPA and MVPA. Abbreviations: SED, sedentary behavior; PROSED, prolonged sedentary behavior; STAND, standing behavior; TPA, total physical activity; LPA, light intensity physical activity; MVPA, moderate- to-vigorous physical activity; I<O, dichotomy index.

* Models were already adjusted for sex (male/female), age at enrollment (years), time since end of treatment (days), neo-adjuvant chemotherapy and/or radiotherapy (yes/no), adjuvant therapy chemotherapy (yes/no), comorbidities (0, 1, ≥2), BMI (kg/m^2), stoma (yes/no), smoking (former, current, never), employment status (yes/no), education level (low/medium/high) and partner status (yes/no).

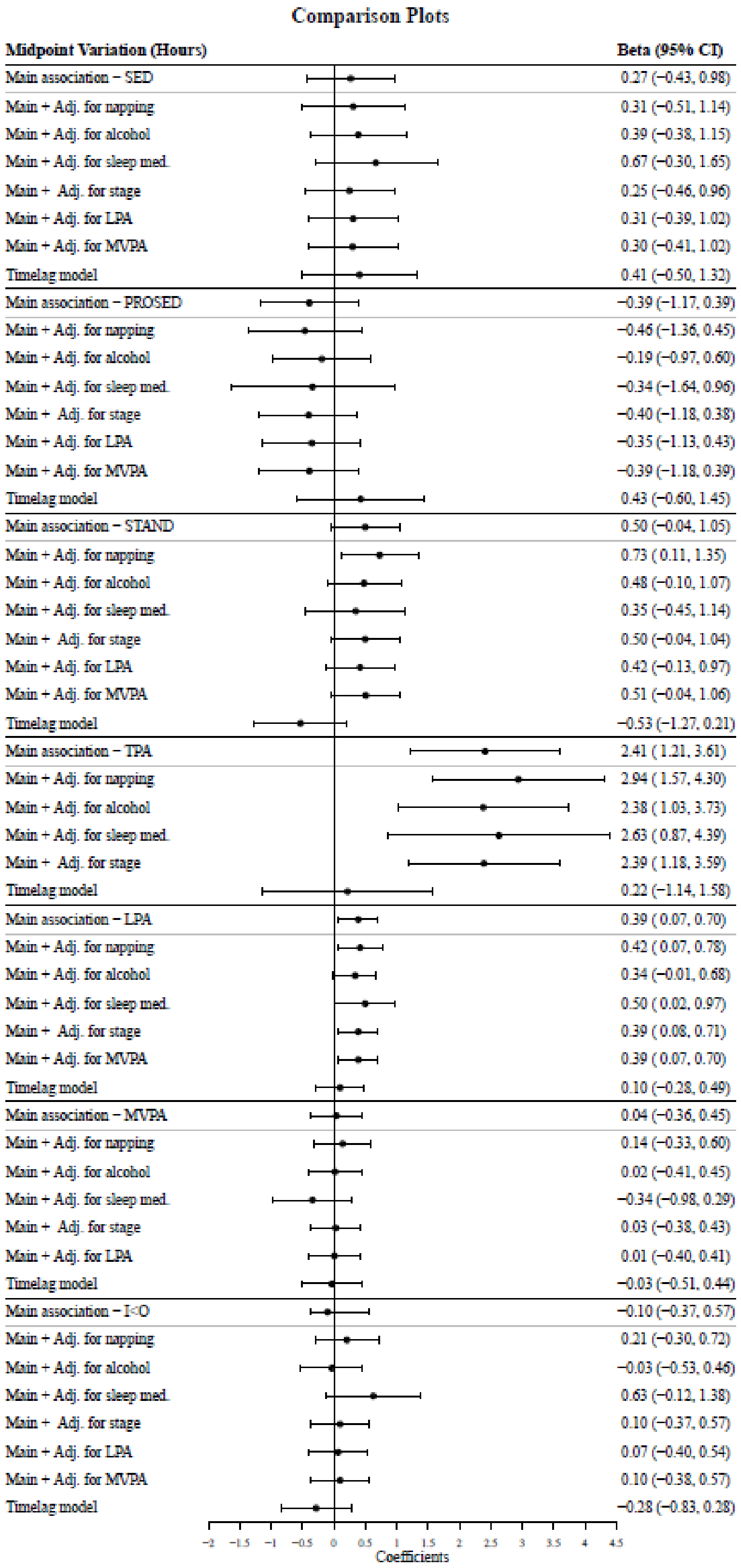

Supplementary Figure 5. Longitudinal adjusted associations of the various sensitivity analysis comparing the main associations with the outcome midpoint of sleep variation with the additional adjustment to the main model for napping, alcohol, sleep medication, stage, LPA and MVPA. Abbreviations: SED, sedentary behavior; PROSED, prolonged sedentary behavior; STAND, standing behavior; TPA, total physical activity; LPA, light intensity physical activity; MVPA, moderate-to-vigorous physical activity; I<O, dichotomy index.

* Models were already adjusted for sex (male/female), age at enrollment (years), time since end of treatment (days), neo-adjuvant chemotherapy and/or radiotherapy (yes/no), adjuvant therapy chemotherapy (yes/no), comorbidities (0, 1, ≥2), BMI (kg/m^2), stoma (yes/no), smoking (former, current, never), employment status (yes/no), education level (low/medium/high) and partner status (yes/no).

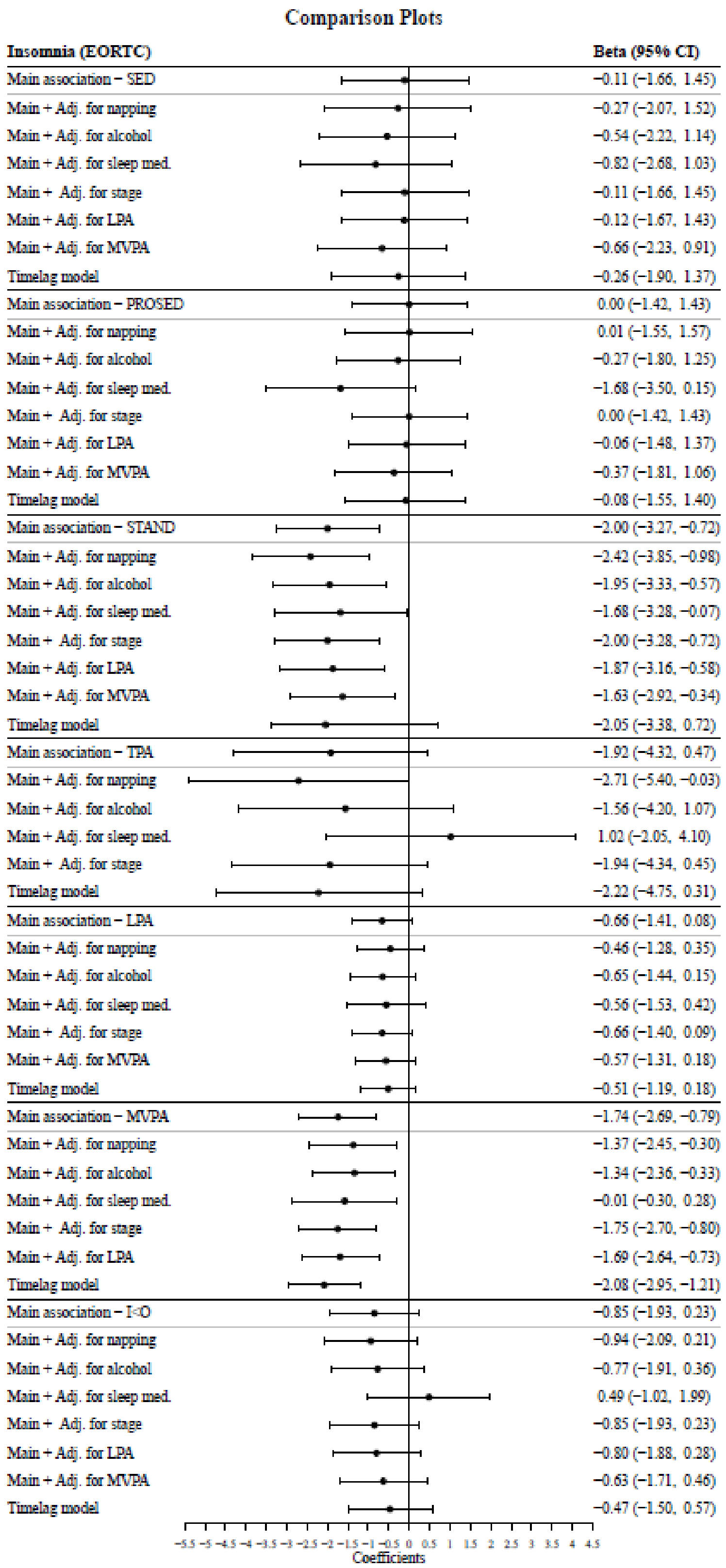

Supplementary Figure 6. Longitudinal adjusted associations of the various sensitivity analysis comparing the main associations with the outcome insomnia with the additional adjustment to the main model for napping, alcohol, sleep medication, stage, LPA and MVPA. Abbreviations: SED, sedentary behavior; PROSED, prolonged sedentary behavior; STAND, standing behavior; TPA, total physical activity; LPA, light intensity physical activity; MVPA, moderate- to-vigorous physical activity; I<O, dichotomy index.

* Models were already adjusted for sex (male/female), age at enrollment (years), time since end of treatment (days), neo-adjuvant chemotherapy and/or radiotherapy (yes/no), adjuvant therapy chemotherapy (yes/no), comorbidities (0, 1, ≥2), BMI (kg/m^2), stoma (yes/no), smoking (former, current, never), employment status (yes/no), education level (low/medium/high) and partner status (yes/no).

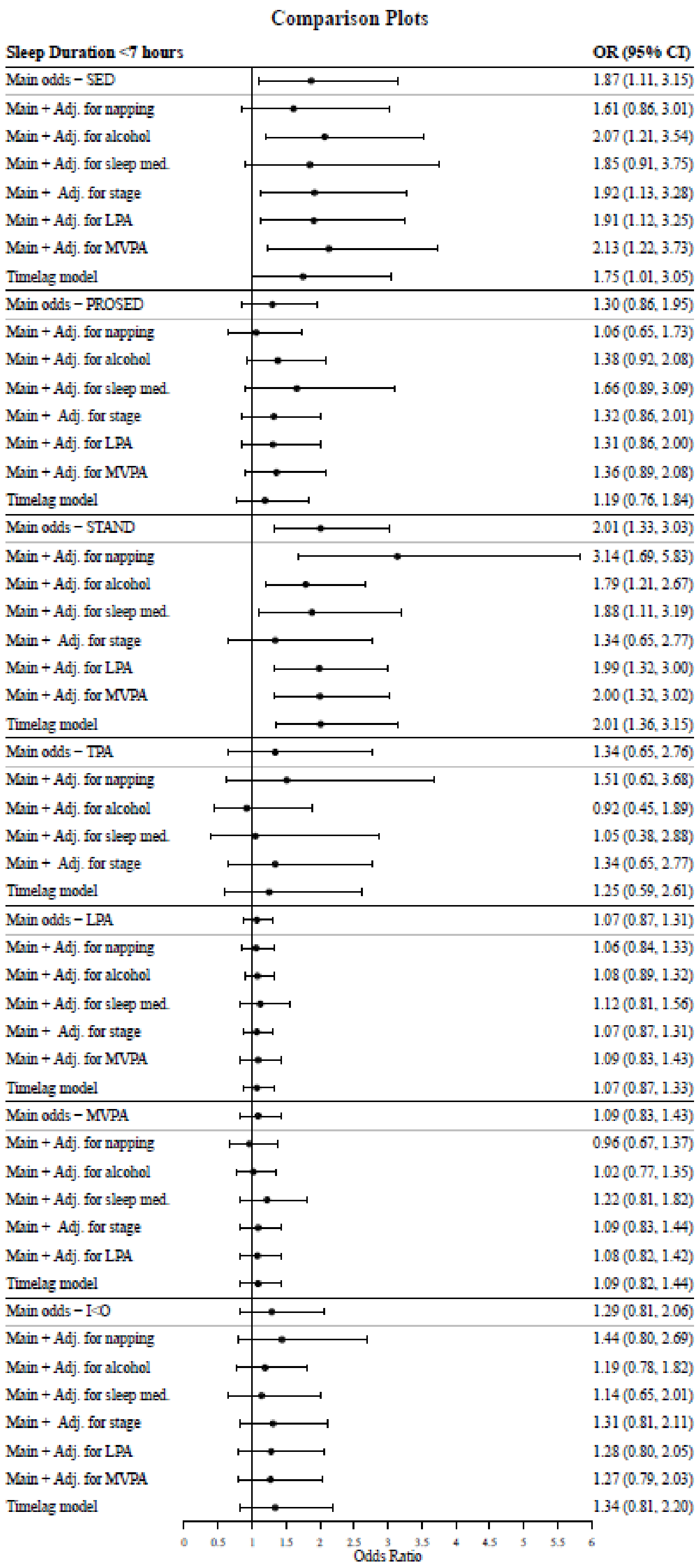

Supplementary Figure 7. Longitudinal adjusted associations of the various sensitivity analysis comparing the main odds ratio of ending up in sleep duration <7 hours with additional adjustment to the main model for napping, alcohol, sleep medication, stage, LPA and MVPA. Abbreviations: SED, sedentary behavior; PROSED, prolonged sedentary behavior; STAND, standing behavior; TPA, total physical activity; LPA, light intensity physical activity; MVPA, moderate- to-vigorous physical activity; I<O, dichotomy index.

* Models were already adjusted for sex (male/female), age at enrollment (years), time since end of treatment (days), neo-adjuvant chemotherapy and/or radiotherapy (yes/no), adjuvant therapy chemotherapy (yes/no), comorbidities (0, 1, ≥2), BMI (kg/m^2), stoma (yes/no), smoking (former, current, never), employment status (yes/no), education level (low/medium/high) and partner status (yes/no).

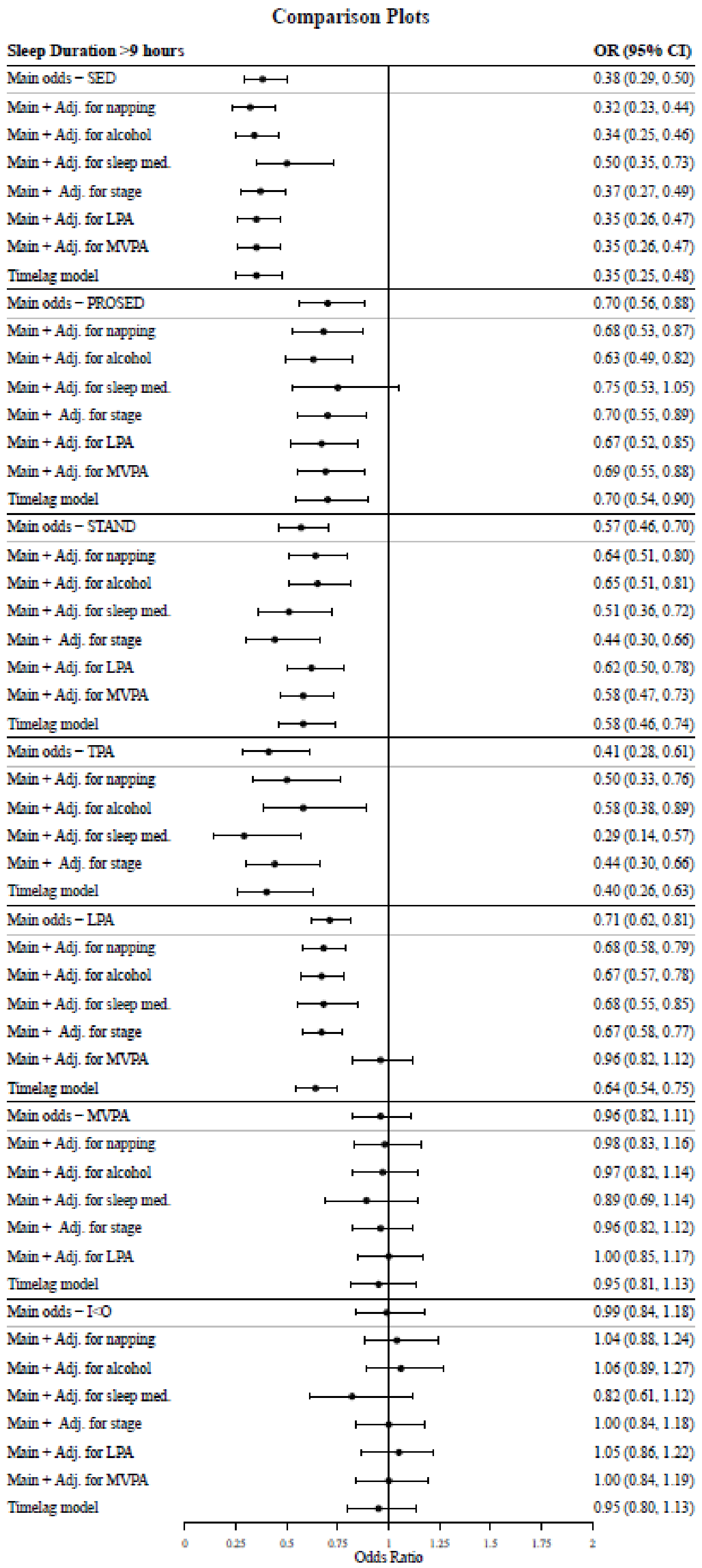

Supplementary Figure 8. Longitudinal adjusted associations of the various sensitivity analysis comparing the main odds ratio of ending up in sleep duration >9 hours with additional adjustment to the main model for napping, alcohol, sleep medication, stage, LPA and MVPA. Abbreviations: SED, sedentary behavior; PROSED, prolonged sedentary behavior; STAND, standing behavior; TPA, total physical activity; LPA, light intensity physical activity; MVPA, moderate- to-vigorous physical activity; I<O, dichotomy index.

* Models were already adjusted for sex (male/female), age at enrollment (years), time since end of treatment (days), neo-adjuvant chemotherapy and/or radiotherapy (yes/no), adjuvant therapy chemotherapy (yes/no), comorbidities (0, 1, ≥2), BMI (kg/m^2), stoma (yes/no), smoking (former, current, never), employment status (yes/no), education level (low/medium/high) and partner status (yes/no).

Supplementary Table 9. Sensitivity analysis comparing associations up to 24 months versus up to 60 months post-treatment. *Values in bold are statistically significant (P < 0.05).*

		**Sleep Duration** Mean sleep duration in hours per night (h/night)		**Coefficient of sleep variation** Variation in sleep duration (0-100%)		**Coefficient of midpoint of sleep** Variation in midpoint of sleep (0-100%)		**Sleep quality** PSQI global score (0-21 points)		**Insomnia** EORTC-QLQ-C30 (0-100 score)	
		β[1]	95% CI	β[1]	95% CI	β[1]	95% CI	β[1]	95% CI	β[1]	95% CI
**Total sedentary behavior (2h/day)** *(MOX)*	Up to M60	**-0.28**	**(-0.35, -0.21)**	0.11	(-0.26, 0.48)	0.27	(-0.43, 0.98)	0.09	(-0.13, 0.31)	-0.11	(-1.66, 1.45)
	Up to M24	**-0.27**	**(-0.35, -0.20)**	0.13	(-0.26, 0.53)	0.24	(-0.53, 1.00)	-0.03	(-0.27, 0.21)	-0.30	(-1.94, 1.35)
**Prolonged sedentary behavior (2h/day)** *(MOX)*	Up to M60	-0.06	(-0.11, 0.00)	-0.08	(-0.34, 0.21)	-0.39	(-1.17, 0.39)	0.12	(-0.10, 0.35)	0.00	(-1.42, 1.43)
	Up to M24	-0.05	(-0.11, 0.01)	-0.07	(-0.37, 0.24)	-0.46	(-1.19, 0.27)	-0.02	(-0.27, 0.22)	-0.06	(-1.54, 1.42)
**Standing behavior (h/day)** *(MOX)*	Up to M60	**-0.21**	**(-0.25, -0.16)**	**0.29**	**(0.03, 0.55)**	0.50	(-0.04, 1.05)	**-0.24**	**(-0.43, -0.04)**	**-2.00**	**(-3.27, -0.72)**
	Up to M24	**-0.21**	**(-0.26, -0.16)**	**0.27**	**(-0.00, 0.55)**	0.56	(-0.01, 1.12)	-0.21	(-0.42, 0.00)	**-2.06**	**(-3.39, -0.72**
**Total physical activity (h/day)** *(MOX)*	Up to M60	**-0.31**	**(-0.41, -0.21)**	**0.59**	**(0.11, 1.10)**	**2.41**	**(1.21, 3.61)**	-0.26	(-0.64, 0.12)	-1.92	(-4.32, 0.47)
	Up to M24	**-0.32**	**(-0.42, -0.21)**	**0.63**	**(0.11, 1.15)**	**2.62**	**(1.34, 3.90)**	-0.19	(-0.61, 0.24)	-2.28	(-4.82, 0.26)
**Light-intensity physical activity (h/day)** *(Self-reported SQUASH)*	Up to M60	**-0.07**	**(-0.10, -0.05)**	0.05	(-0.10, 0.20)	**0.39**	**(0.07, 0.70)**	-0.06	(-0.18, 0.06)	-0.66	(-1.41, 0.08)
	Up to M24	**-0.07**	**(-0.10, -0.05)**	0.04	(-0.12, 0.21)	**0.35**	**(0.01, 0.70)**	-0.09	(-0.23, 0.04)	-0.50	(-1.28, 0.28)
**Moderate-to-vigorous-intensity physical activity (h/day)** *(Self-reported SQUASH)*	Up to M60	**-0.04**	**(-0.07, -0.00)**	-0.06	(-0.25, 0.13)	0.04	(-0.36, 0.45)	-0.12	(-0.28, 0.04)	**-1.74**	**(-2.69, -0.79)**
	Up to M24	**-0.04**	**(-0.07, -0.00)**	-0.08	(-0.29, 0.12)	0.07	(-0.36, 0.49)	-0.07	(-0.25, 0.11)	**-1.77**	**(-2.77, -0.78)**
**Dichotomy index[2]** *(MOX)*	Up to M60	-0.03	(-0.07, 0.00)	-0.08	(-0.31, 0.15)	-0.10	(-0.37, 0.57)	0.02	(-0.15, 0.19)	-0.85	(-1.93, 0.23)
	Up to M24	**-0.04**	**(-0.07, -0.00)**	-0.09	(-0.32, 0.15)	-0.13	(-0.36, 0.63)	0.05	(-0.13, 0.23)	-0.72	(-1.82, 0.38)

[1] The β-coefficients indicate the overall longitudinal difference in the outcome score using linear mixed models per 2 hours increase in sedentary behavior variables, per 1 hour increase in physical activity variables or per 1 SD increase in the dichotomy index.

[2]The dichotomy index was included in the model per standard deviation (SD) increase, being the average SD across time points.

**Linear mixed-models were adjusted for sex (male/female), age at enrollment (years), time since end of treatment (days), neo-adjuvant chemotherapy and/or radiotherapy (yes/no), adjuvant therapy chemotherapy (yes/no), comorbidities (0, 1, ≥2), BMI (kg/m^2), stoma (yes/no), smoking (former, current, never), employment status (yes/no), education level (low/medium/high) and partner status (yes/no).*

	**Sleep Duration (Up to 60 months)**	**Sleep Duration (Up to 24 months)**

		OR[1]	95% CI of OR	OR[1]	95% CI of OR
**Total sedentary behavior (2h/day)** *(MOX)*	<7 hours	**1.87**	**(1.11, 3.15)**	1.73	(0.99, 3.00)
	7-9 hours (REF)				
	>9 hours	**0.38**	**(0.29, 0.50)**	**0.34**	**(-0.25, 0.48)**
**Prolonged sedentary behavior (2h/day)** *(MOX)*	<7 hours	1.30	(0.86, 1.95)	1.17	(0.76, 1.83)
	7-9 hours (REF)				
	>9 hours	**0.70**	**(0.56, 0.88)**	**0.69**	**(0.53, 0.89)**
**Standing behavior (h/day)** *(MOX)*	<7 hours	**2.01**	**(1.33, 3.03)**	**2.10**	**(1.38, 3.20)**
	7-9 hours (REF)				
	>9 hours	**0.57**	**(0.46, 0.70)**	**0.58**	**(0.46, 0.73)**
**Total physical activity (h/day)** *(MOX)*	<7 hours	1.34	(0.65, 2.76)	1.25	(0.60, 2.61)
	7-9 hours (REF)				
	>9 hours	**0.41**	**(0.28, 0.61)**	**0.41**	**(0.26, 0.64)**
**Light-intensity physical activity (h/day)** *(Self-reported SQUASH)*	<7 hours	1.07	(0.87, 1.31)	1.07	(0.86, 1.32)
	7-9 hours (REF)				
	>9 hours	**0.71**	**(0.62, 0.81)**	**0.63**	**(0.53, 0.74)**
**Moderate-to-vigorous-intensity-physical activity (h/day)** *(Self-reported SQUASH)*	<7 hours	1.09	(0.83, 1.43)	1.10	(0.83, 1.46)
	7-9 hours (REF)				
	>9 hours	0.96	(0.82, 1.11)	0.96	(0.81, 1.14)
**Dichotomy index** *(MOX)*	<7 hours	1.29	(0.81, 2.06)	1.34	(0.82, 2.21)
	7-9 hours (REF)				
	>9 hours	0.99	(0.84, 1.18)	0.99	(0.83, 1.19)

Supplementary Table 10. Sensitivity analysis comparing associations up to 24 months versus up to 60 months post-treatment.
Values in bold are statistically significant (P < 0.05).
[1] The OR indicates the overall odds of being in each sleep duration category relative to the reference category of a normal sleep duration of (7-9 hours of sleep per night) for an increase of 2 hours in sedentary behavior variables, per 1 hour increase in physical activity variables, and per 1 SD increase in the dichotomy index.
REF, reference; OR, odds ratio; the ranges for the various categories are: category 1 (< 7 hours of sleep), category 2 (reference category: 7-9 hours of sleep), category 3 (> 9 hours of sleep).
Abbreviations: SQUASH: Short Questionnaire to Assess Health-enhancing physical activity.
Models were adjusted for sex (male/female), age at enrollment (years), time since end of treatment (days), neo-adjuvant chemotherapy and/or radiotherapy (yes/no), adjuvant therapy chemotherapy (yes/no), comorbidities (0, 1, ≥2), BMI (kg/m²), stoma (yes/no), smoking (former, current, never), employment status (yes/no), education level (low/medium/high) and partner status (yes/no).

	**Sleep Duration** Mean sleep duration in

		hours per night (h/night)	
		β[1]	95% CI
**Total sedentary behavior (2h/day)** *(MOX)*	Adjusted	**-0.28**	**(-0.35, -0.21)**
	Adjusted and additional adjustment for standing	0.04	(-0.00, 0.08)
**Prolonged sedentary behavior (2h/day)** *(MOX)*	Adjusted	-0.06	(-0.11, 0.00)
	Adjusted and additional adjustment for standing	**-0.19**	**(-0.24, -0.13)**
**Total physical activity (h/day)** *(MOX)*	Adjusted	**-0.31**	**(-0.41, -0.21)**
	Adjusted and additional adjustment for standing	**-0.14**	**(-0.25, -0.03)**
**Light-intensity physical activity (h/day)** *(Self-reported SQUASH)*	Adjusted	**-0.07**	**(-0.10, -0.05)**
	Adjusted and additional adjustment for standing	**-0.06**	**(-0.08, -0.03)**

Supplementary Table 11. Sensitivity analysis with an additional adjustment for standing time.
Values in bold are statistically significant (P < 0.05).
[1] *The β-coefficients indicate the overall longitudinal difference in the outcome score using linear mixed models per 2 hours increase in sedentary behavior variables, per 1 hour increase in physical activity variables.*
**Linear mixed-models were adjusted for sex (male/female), age at enrollment (years), time since end of treatment (days), neo-adjuvant chemotherapy and/or radiotherapy (yes/no), adjuvant therapy chemotherapy (yes/no), comorbidities (0, 1, ≥2), BMI (kg/m^2), stoma (yes/no), smoking (former, current, never), employment status (yes/no), education level (low/medium/high) and partner status (yes/no).*

		Sleep Duration		Sleep Duration with additional adjustment for standing
		OR[1]	95% CI of OR	95% CI of OR
**Total sedentary behavior (2h/day)** *(MOX)*	<7 hours	**1.87**	**(1.11, 3.15)**	**9.32 (4.09, 21.21)**
	7-9 hours (REF)			
	>9 hours	**0.38**	**(0.29, 0.50)**	**0.22 (0.16, 0.31)**
**Prolonged sedentary behavior (2h/day)** *(MOX)*	<7 hours	1.30	(0.86, 1.95)	**2.10 (1.25, 3.52)**
	7-9 hours (REF)			
	>9 hours	**0.70**	**(0.56, 0.88)**	**0.48 (0.37, 0.63)**
**Total physical activity (h/day)** *(MOX)*	<7 hours	1.34	(0.65, 2.76)	0.73 (0.33, 1.64)
	7-9 hours (REF)			
	>9 hours	**0.41**	**(0.28, 0.61)**	0.66 (0.42, 1.04)
**Light-intensity physical activity (h/day)** *(Self-reported SQUASH)*	<7 hours	1.07	(0.87, 1.31)	1.04 (0.85, 1.28)
	7-9 hours (REF)			
	>9 hours	**0.71**	**(0.62, 0.81)**	**0.69 (0.60, 0.80)**

Supplementary Table 12. Sensitivity analysis with an additional adjustment for standing time.
Values in bold are statistically significant ($P < 0.05$).
[1] The OR indicates the overall odds of being in each sleep duration category relative to the reference category of a normal sleep duration of (7-9 hours of sleep per night) for an increase of 2 hours in sedentary behavior variables, per 1 hour increase in physical activity variables.
REF, reference; OR, odds ratio; the ranges for the various categories are: category 1 (< 7 hours of sleep), category 2 (reference category: 7-9 hours of sleep), category 3 (> 9 hours of sleep).
Abbreviations: SQUASH: Short Questionnaire to Assess Health-enhancing physical activity.
**Models were adjusted for sex (male/female), age at enrollment (years), time since end of treatment (days), neo-adjuvant chemotherapy and/or radiotherapy (yes/no), adjuvant therapy chemotherapy (yes/no), comorbidities (0, 1, ≥2), BMI (kg/m^2), stoma (yes/no), smoking (former, current, never), employment status (yes/no), education level (low/medium/high) and partner status (yes/no).*
